# Supplementary material for: A phylogenomic framework for charting the diversity and evolution of giant viruses
Source: PLoS Biol. 2021 Oct 27;19(10):e3001430. doi: 10.1371/journal.pbio.3001430 (PMC8575486; doi:10.1371/journal.pbio.3001430)

S1 Text for "A Phylogenomic Framework for Charting the Diversity and Evolution of Giant Viruses**" by Aylward et al.**

Figure A. Major Capsid Protein GVOGm0003 phylogeny.

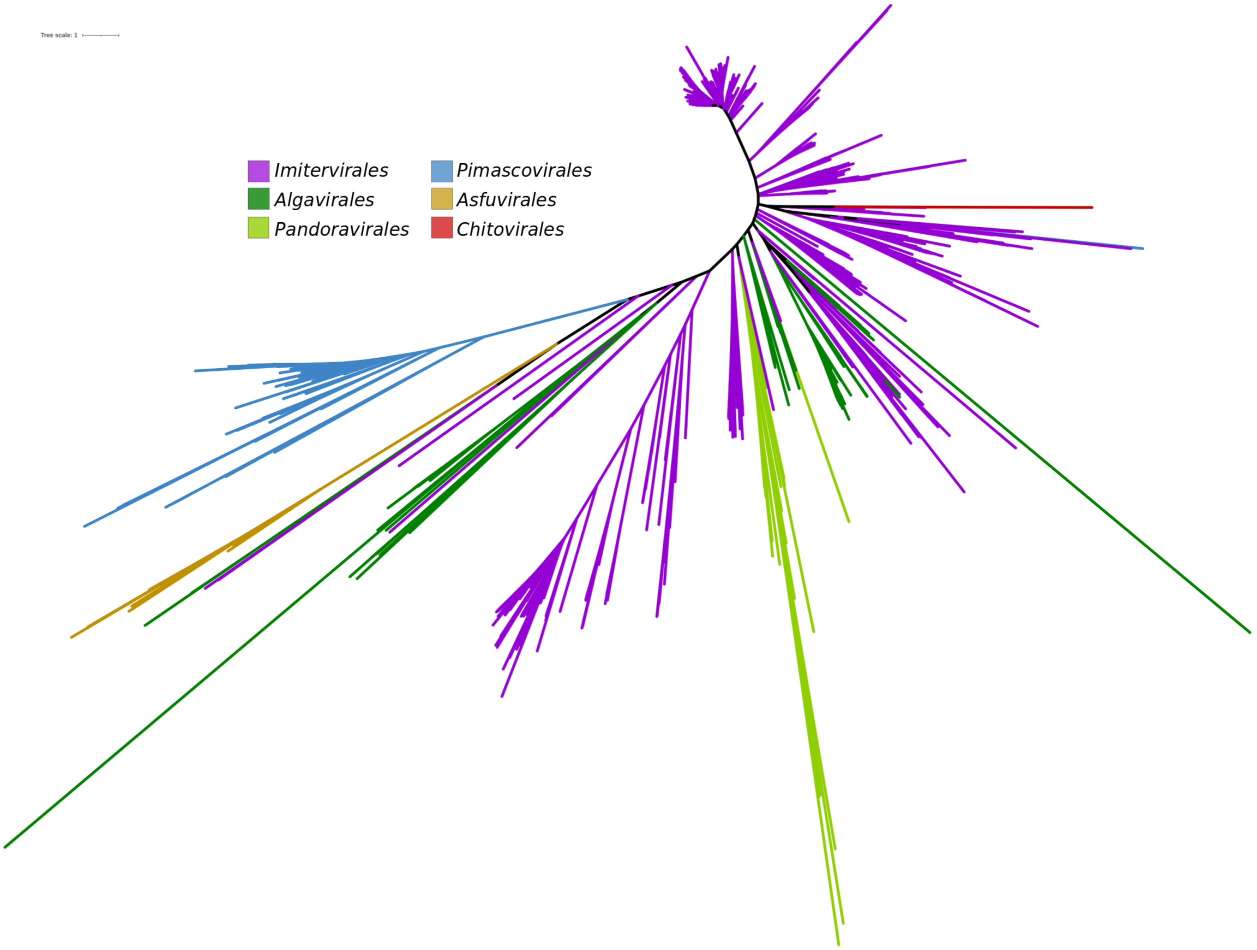

Figure B. Disulfide (thiol) oxidoreductase GVOGm0004 phylogeny.

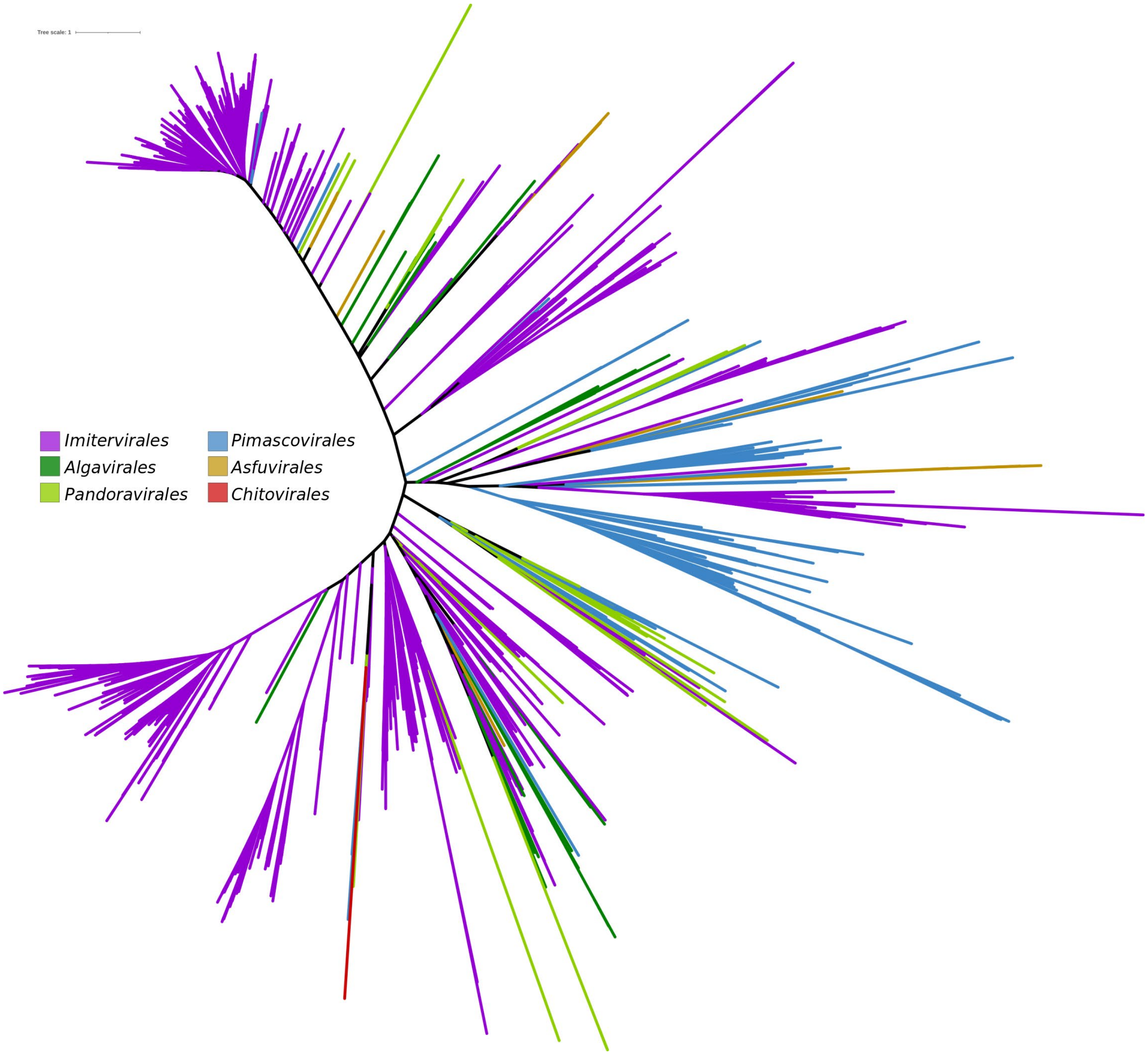

Figure C. Superfamily II helicase GVOGm0013 phylogeny.

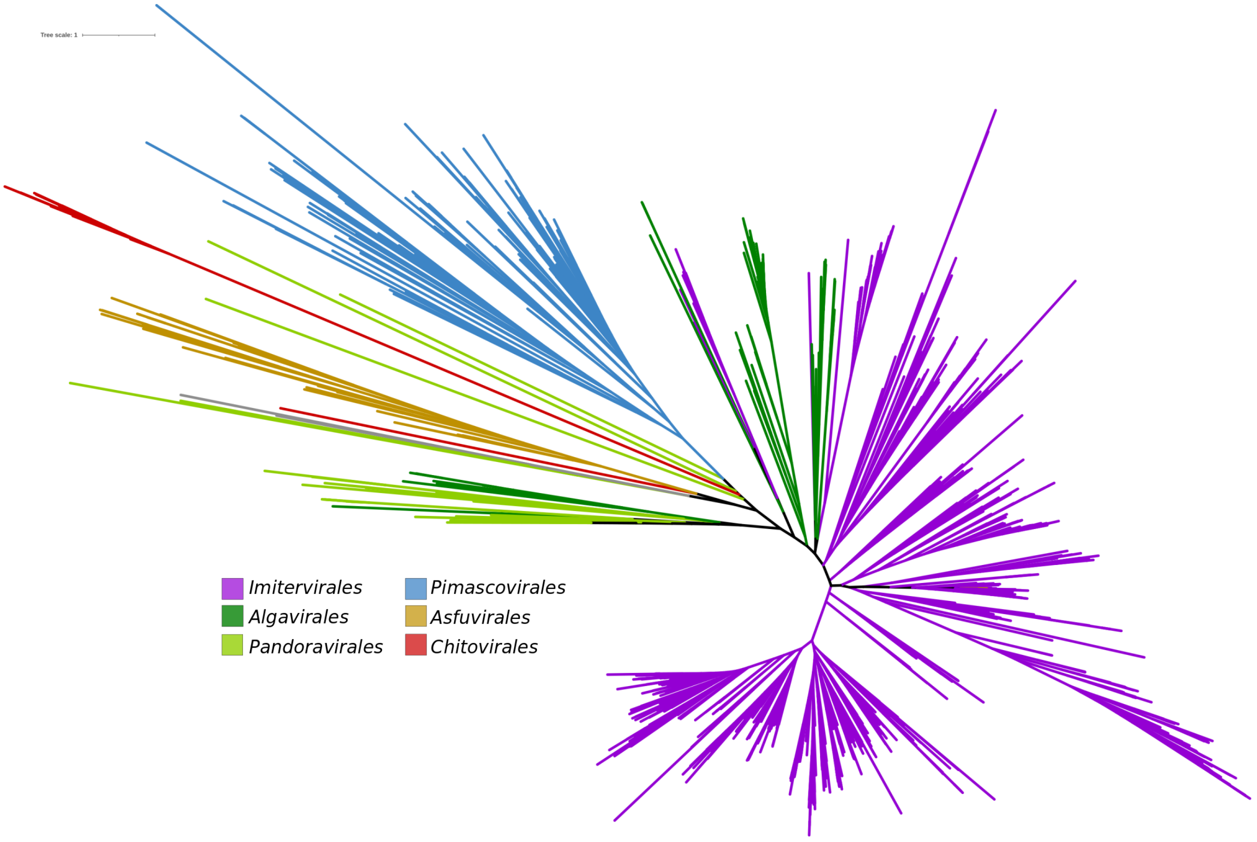

Figure D. Patatin phospholipase GVOGm0018 phylogeny.

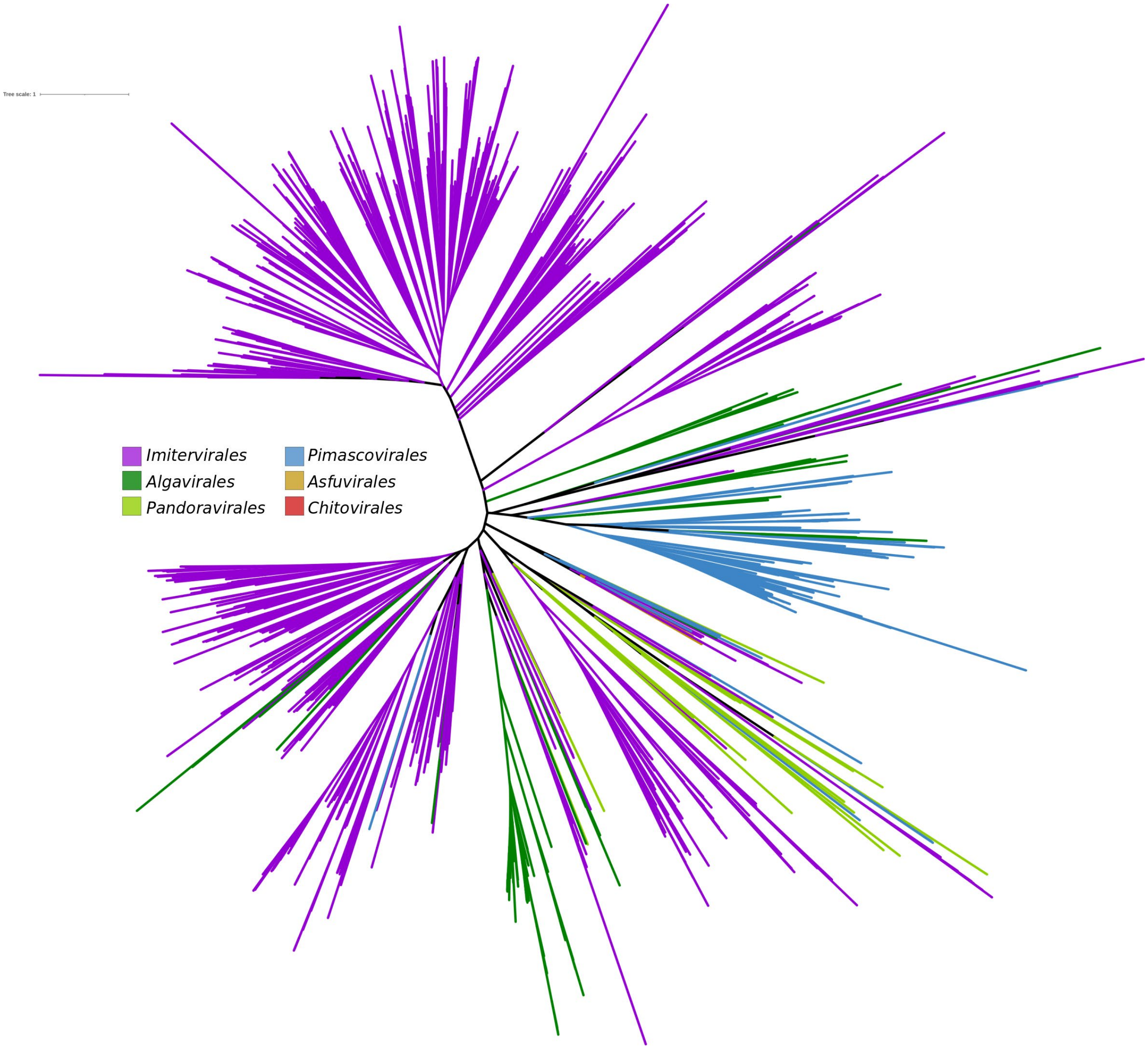

Figure E. DEAD/SNF2-like helicase GVOGm0020 phylogeny.

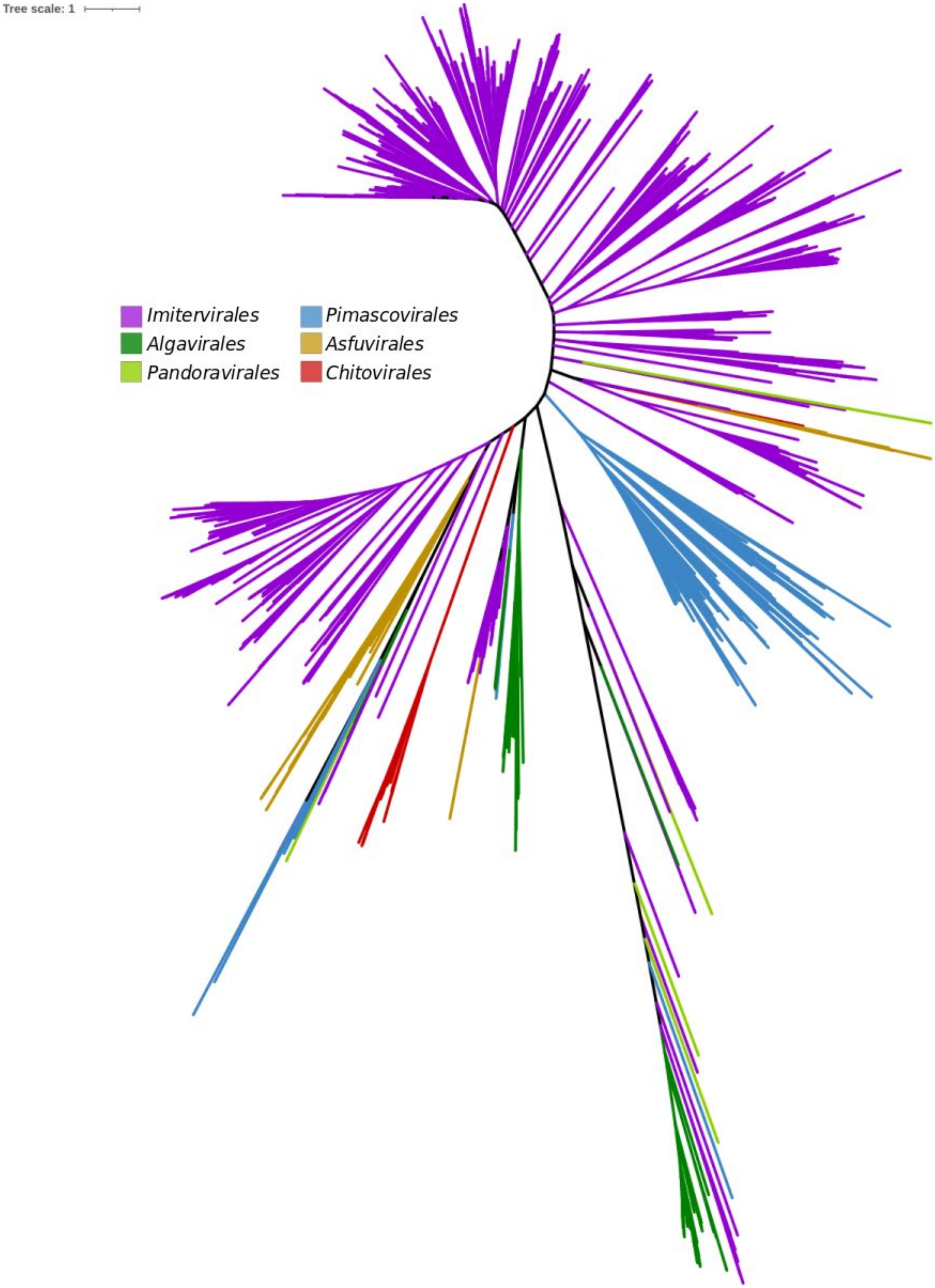

Figure F. DNA-directed RNA polymerase subunit beta (RNAPS) GVOGm0022 phylogeny.

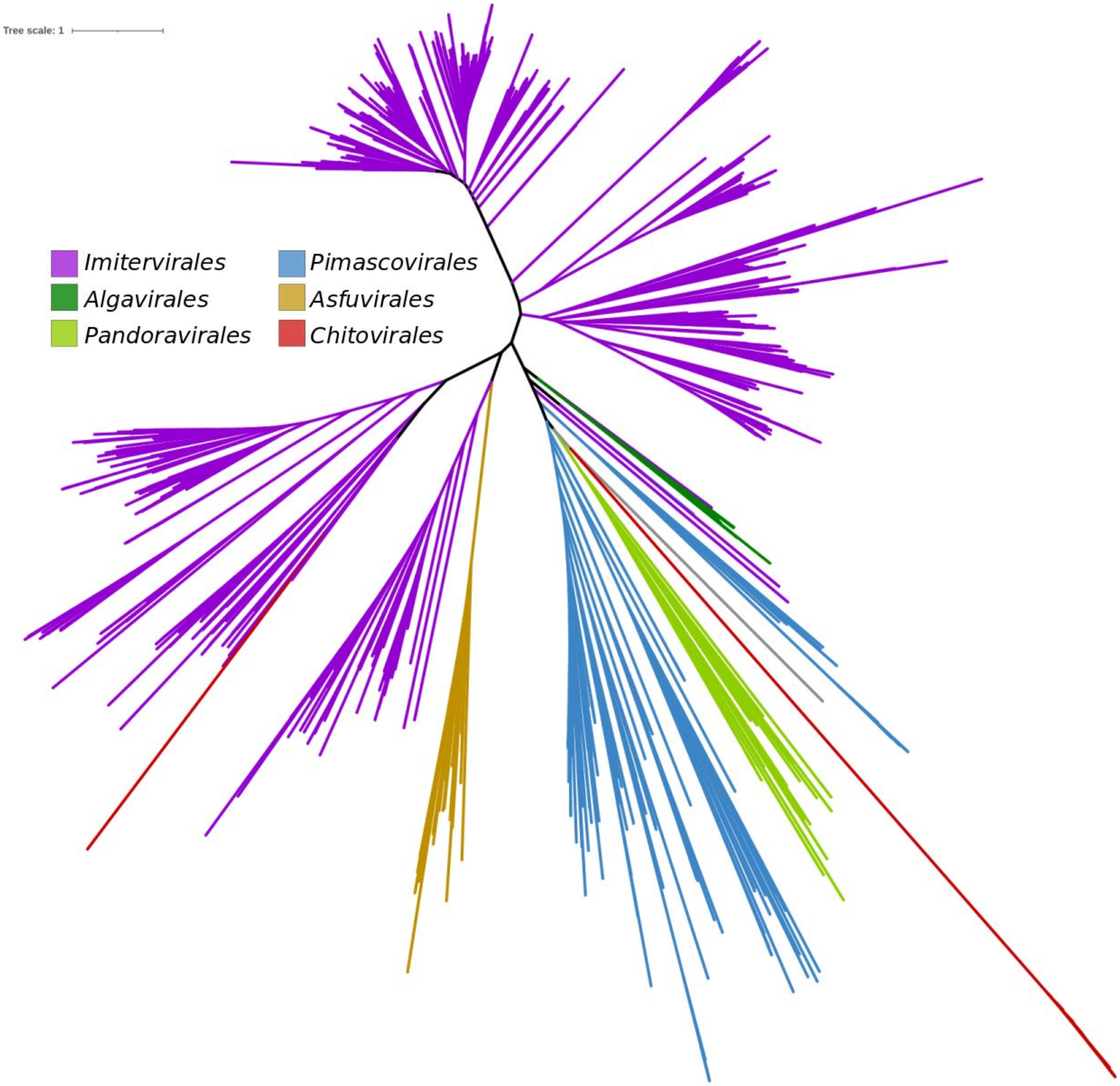

Figure G. DNA-directed RNA polymerase subunit alpha (RNAPL) GVOGm0023 phylogeny.

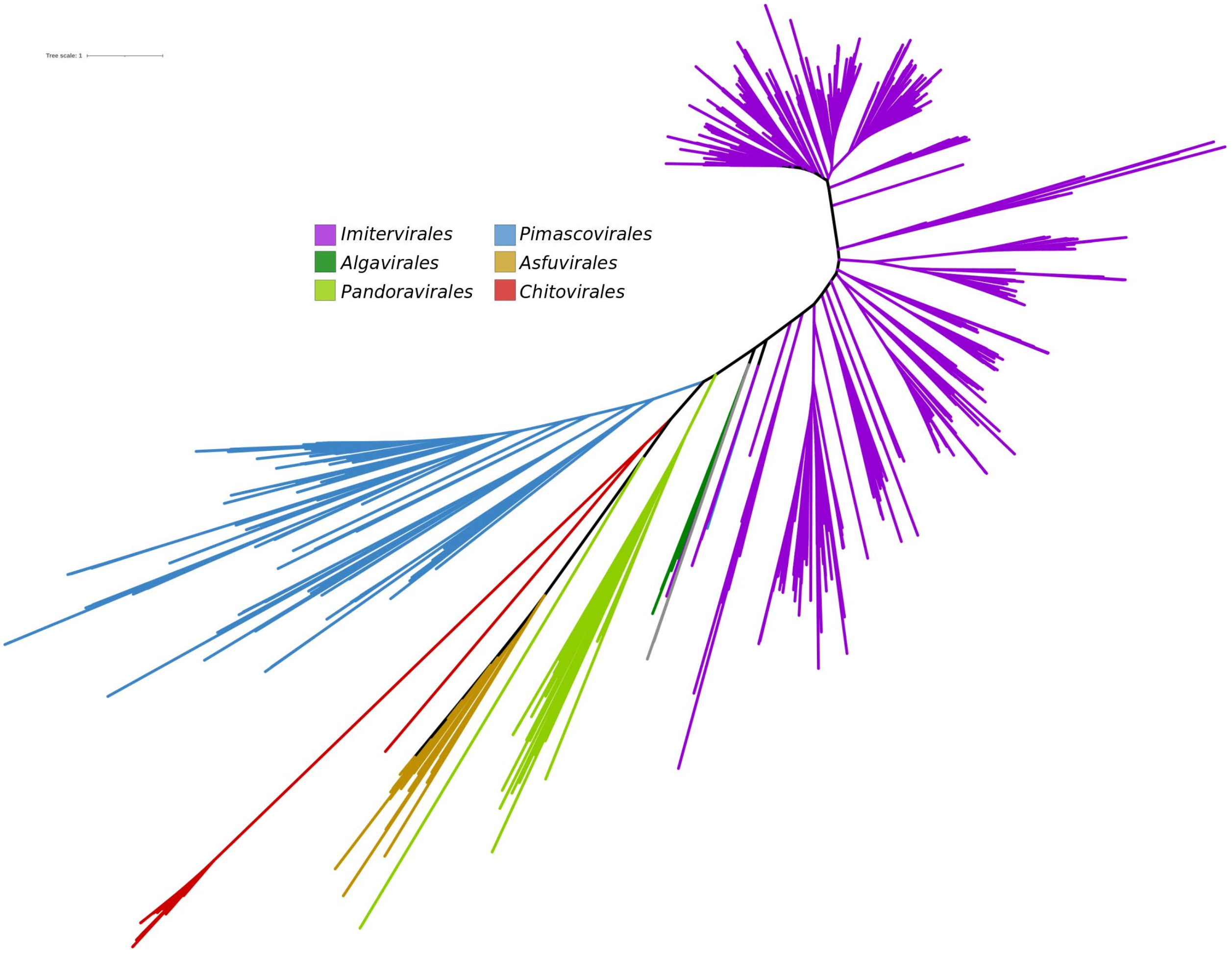

Figure H. mRNA capping enzyme GVOGm0036 phylogeny.

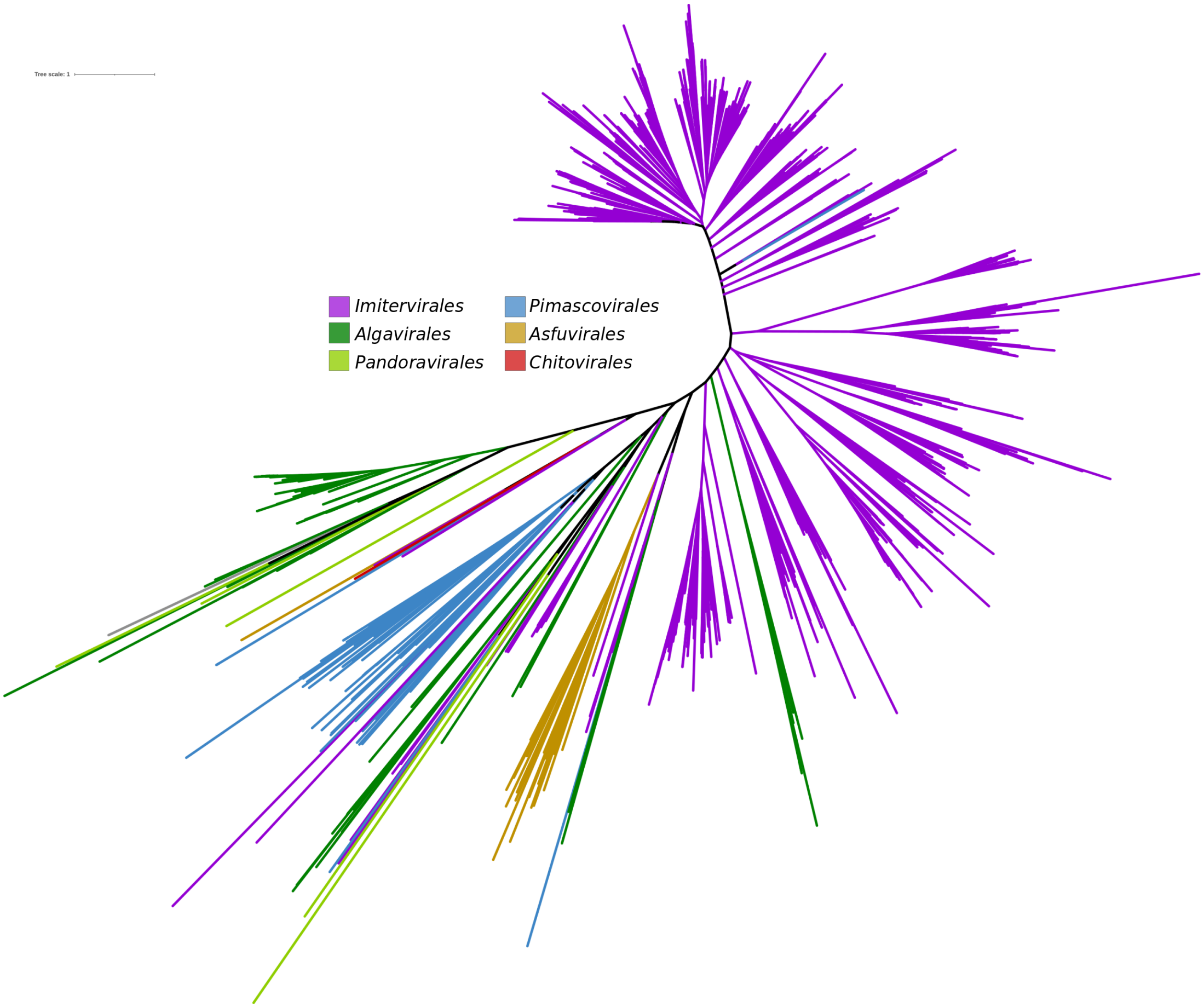

Figure I. DNA polymerase family B GVOGm0054 phylogeny.

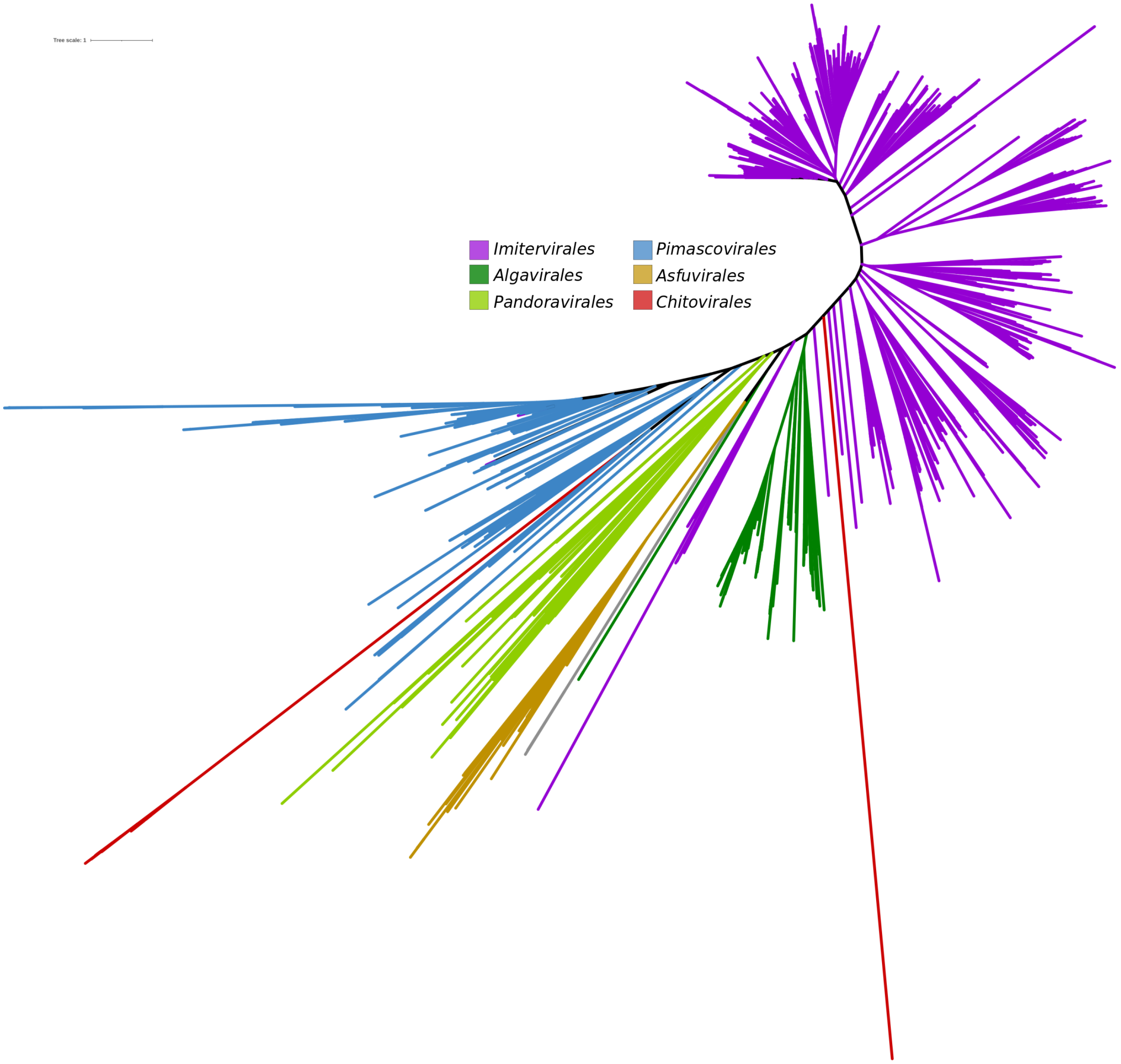

Figure J. TATA box binding protein (TBP) GVOGm0056 phylogeny.

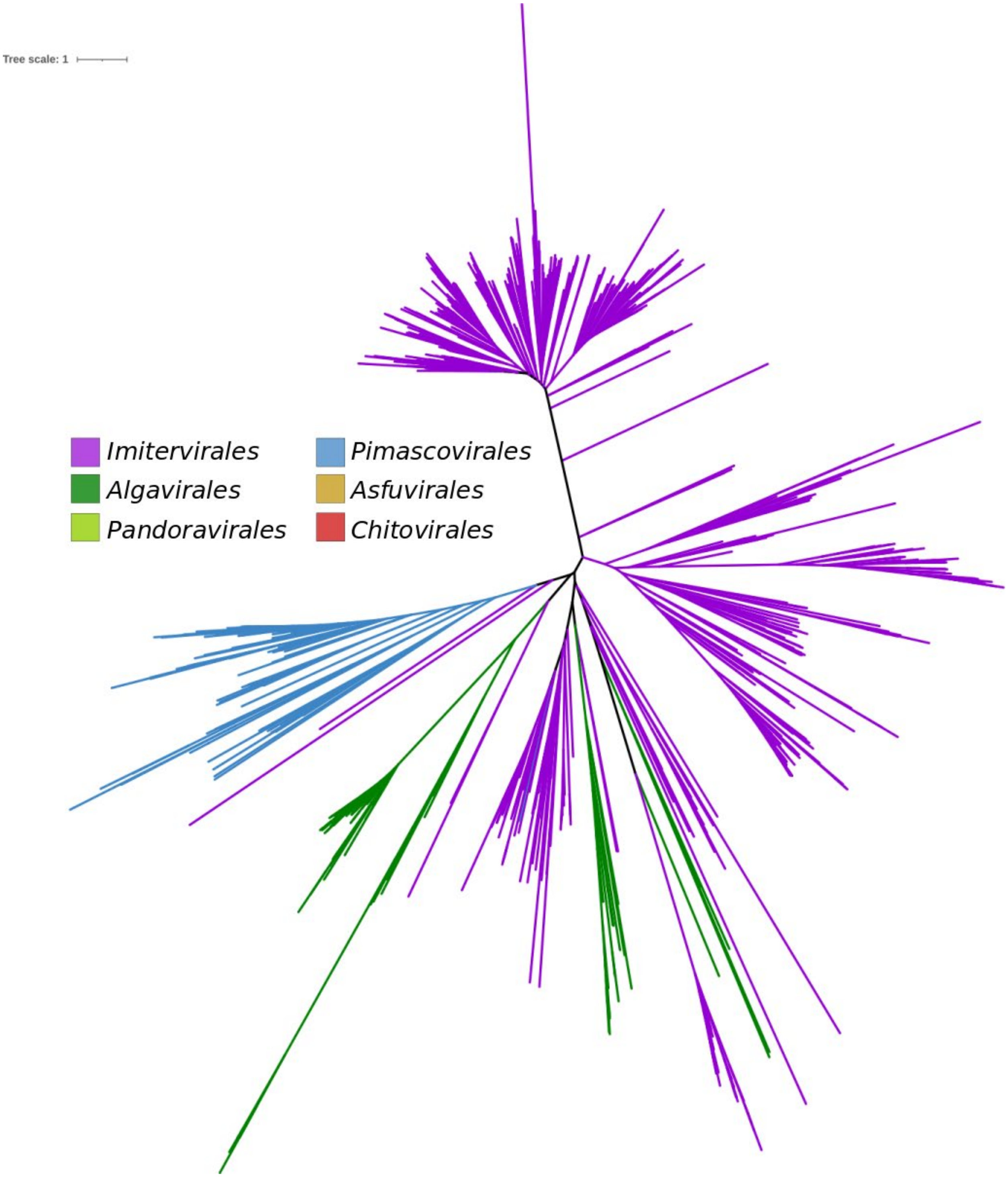

Figure K. Ribonucleoside diphosphate reductase, alpha subunit GVOGm0088 phylogeny.

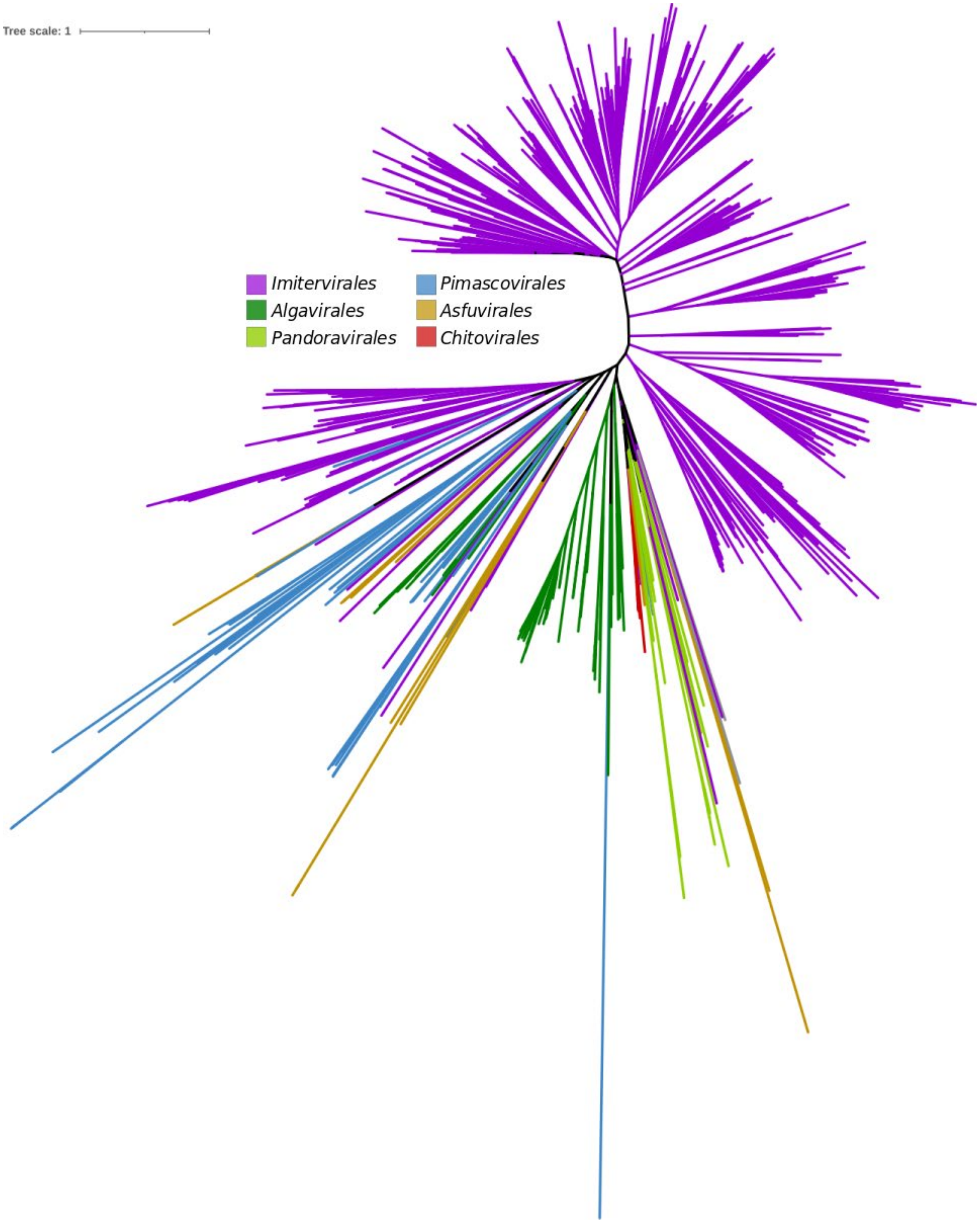

Figure L. D5-like helicase-primase GVOGm0095 phylogeny.

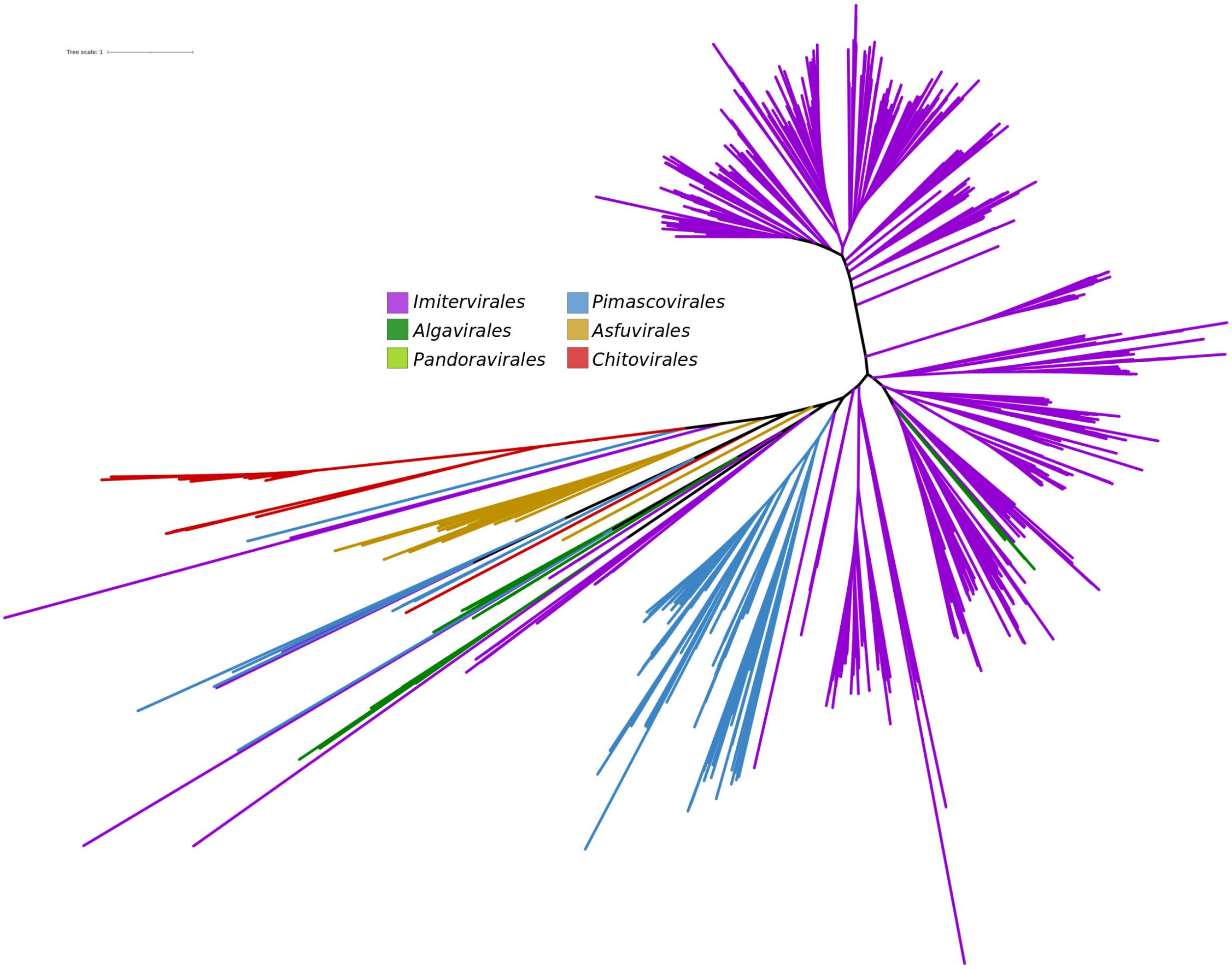

Figure M. Uncharacterized, C-terminal domain GVOGm0115 phylogeny.

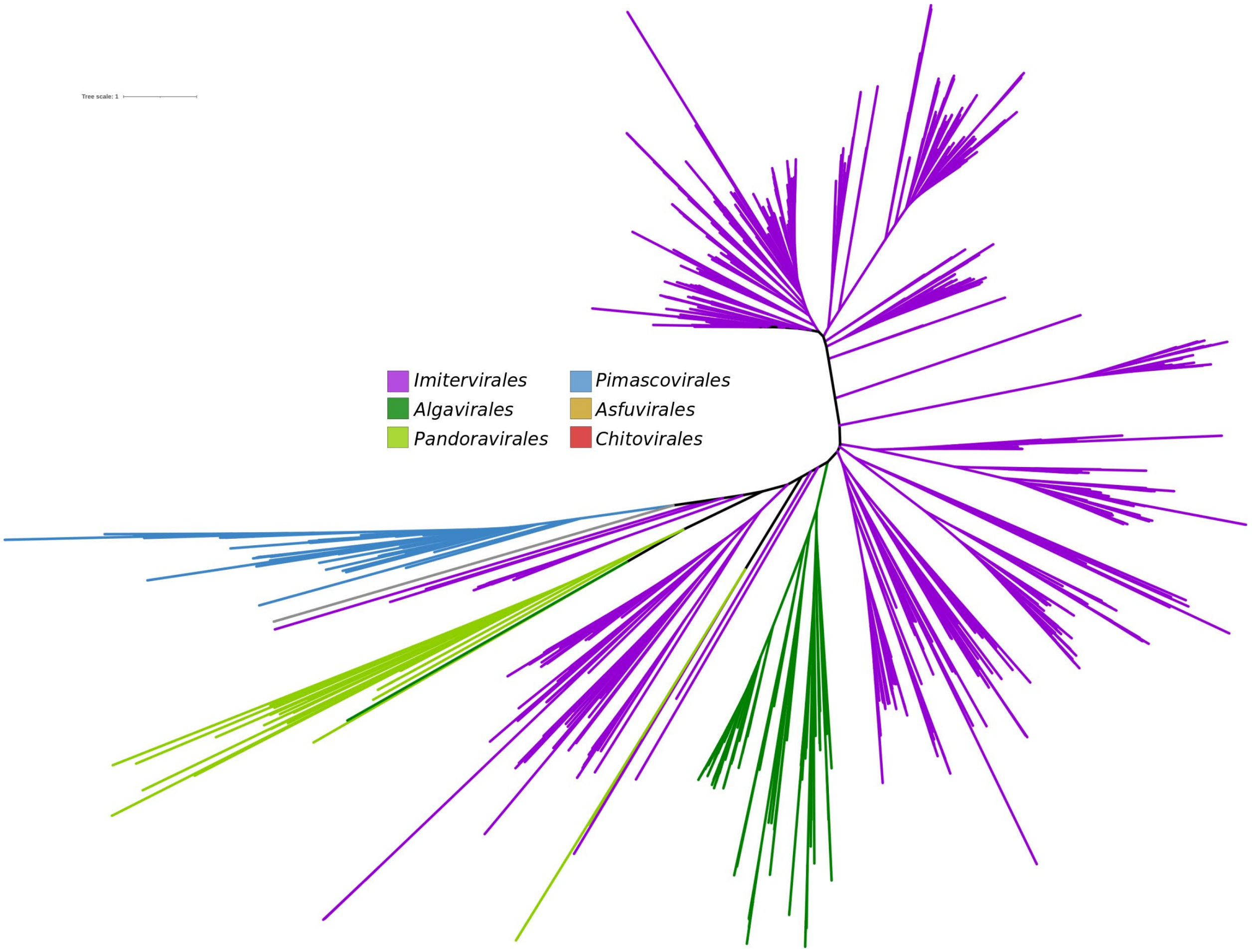

Figure N. Uncharacterized protein GVOGm0152 phylogeny.

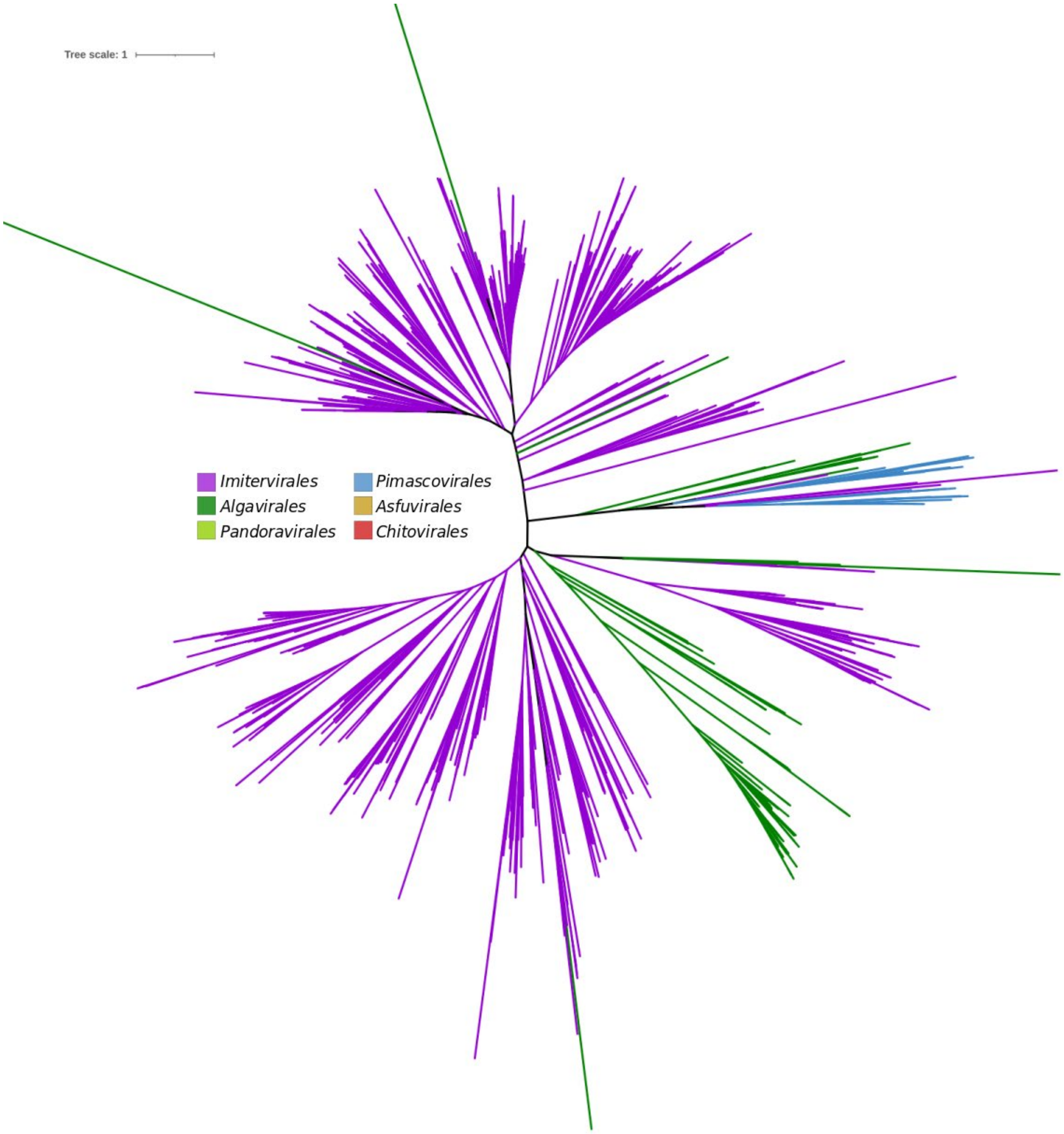

Figure O. Transcription initiation factor IIB GVOGm0172 phylogeny.

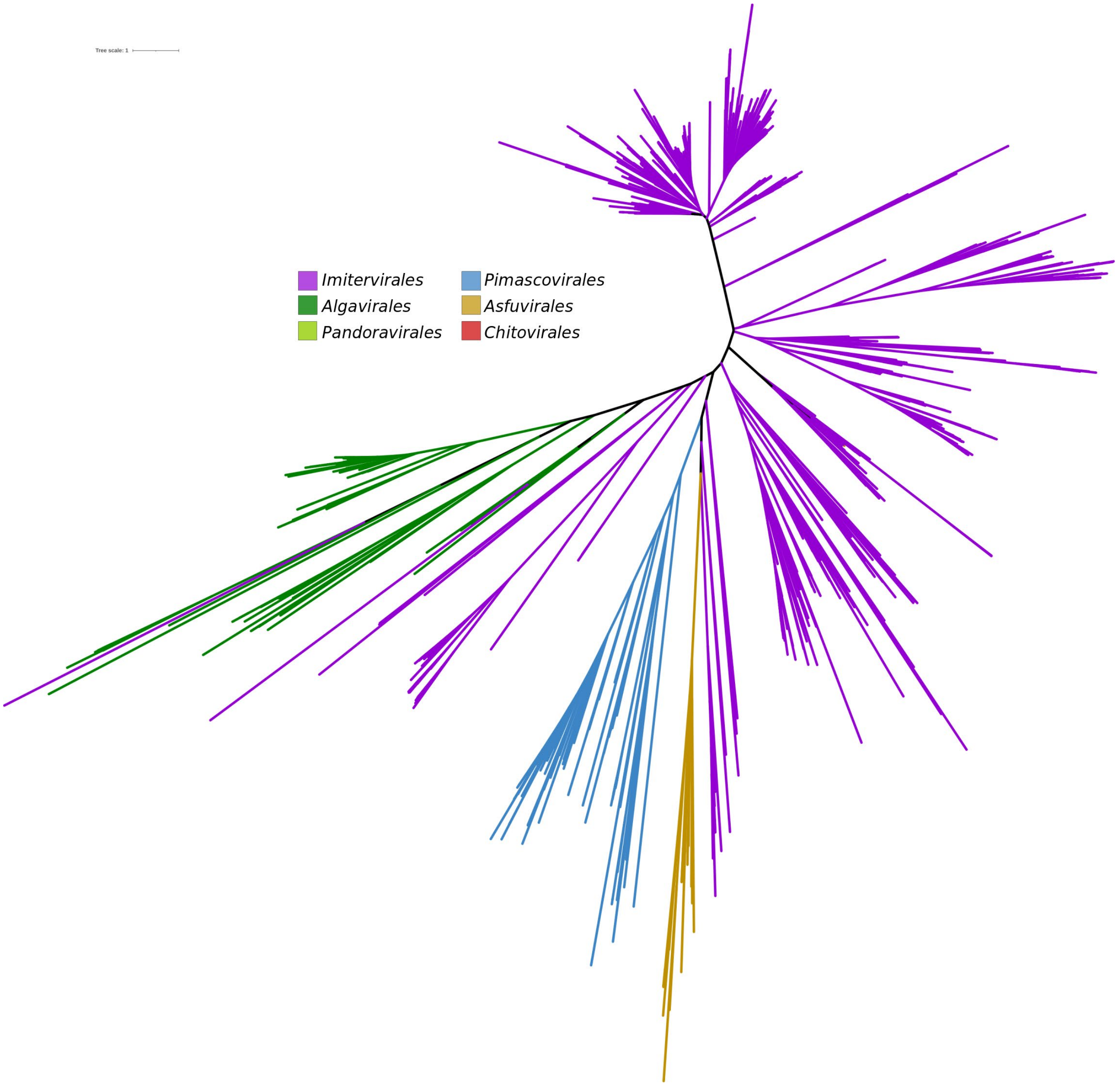

Figure P. RuvC, Holliday junction resolvases (HJRs) GVOGm0189 phylogeny.

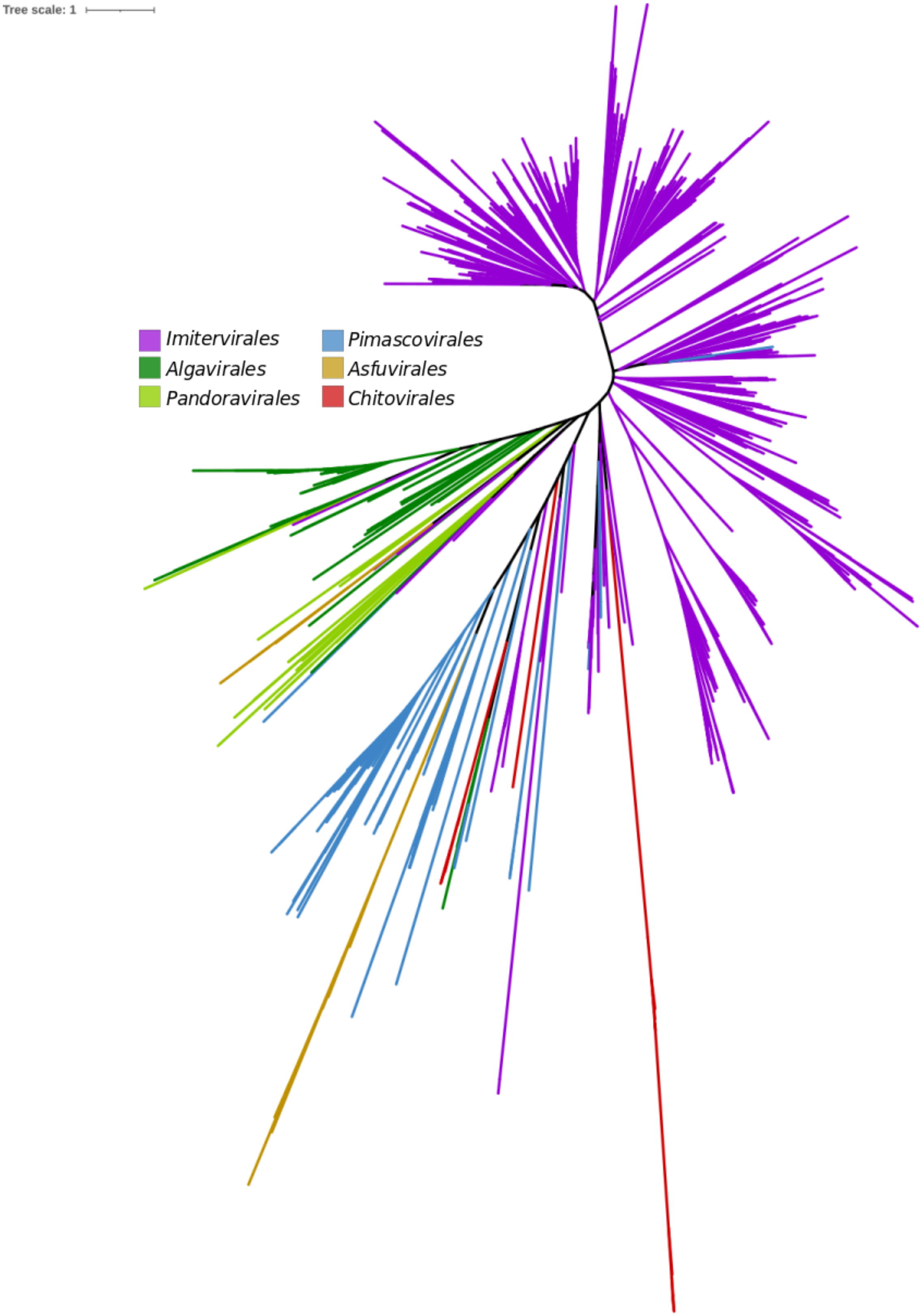

Figure Q. Ubiquitin carboxyl-terminal hydrolase GVOGm0214 phylogeny.

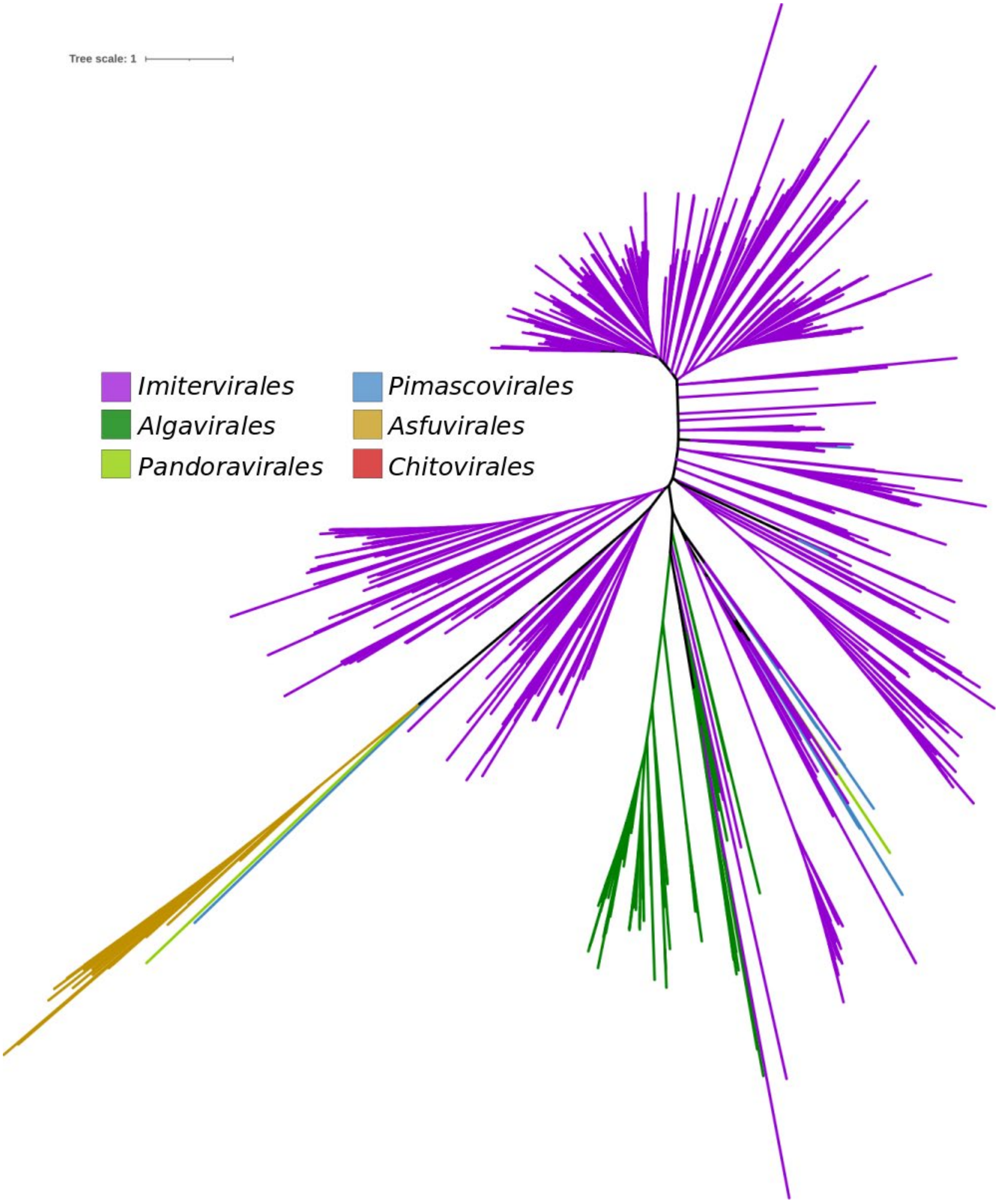

Figure R. Proliferating cell nuclear antigen GVOGm0239 phylogeny.

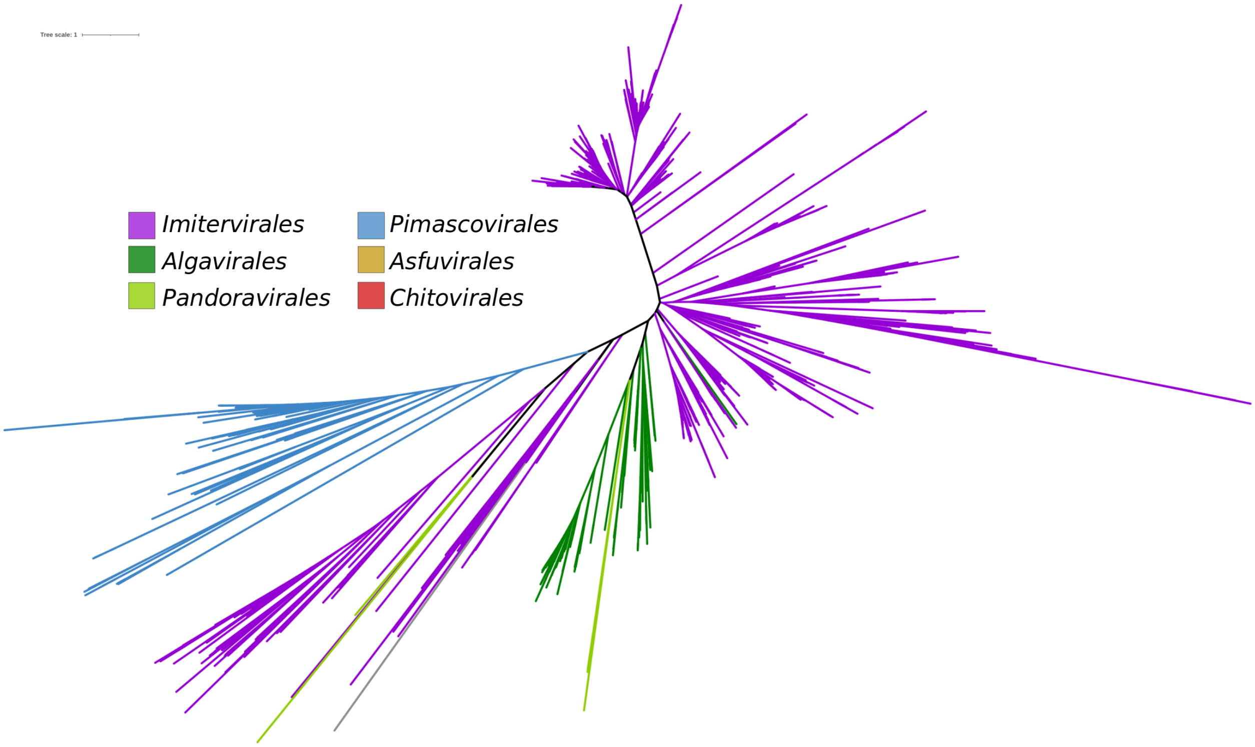

Figure S. DNA topoisomerase II GVOGm0461 phylogeny.

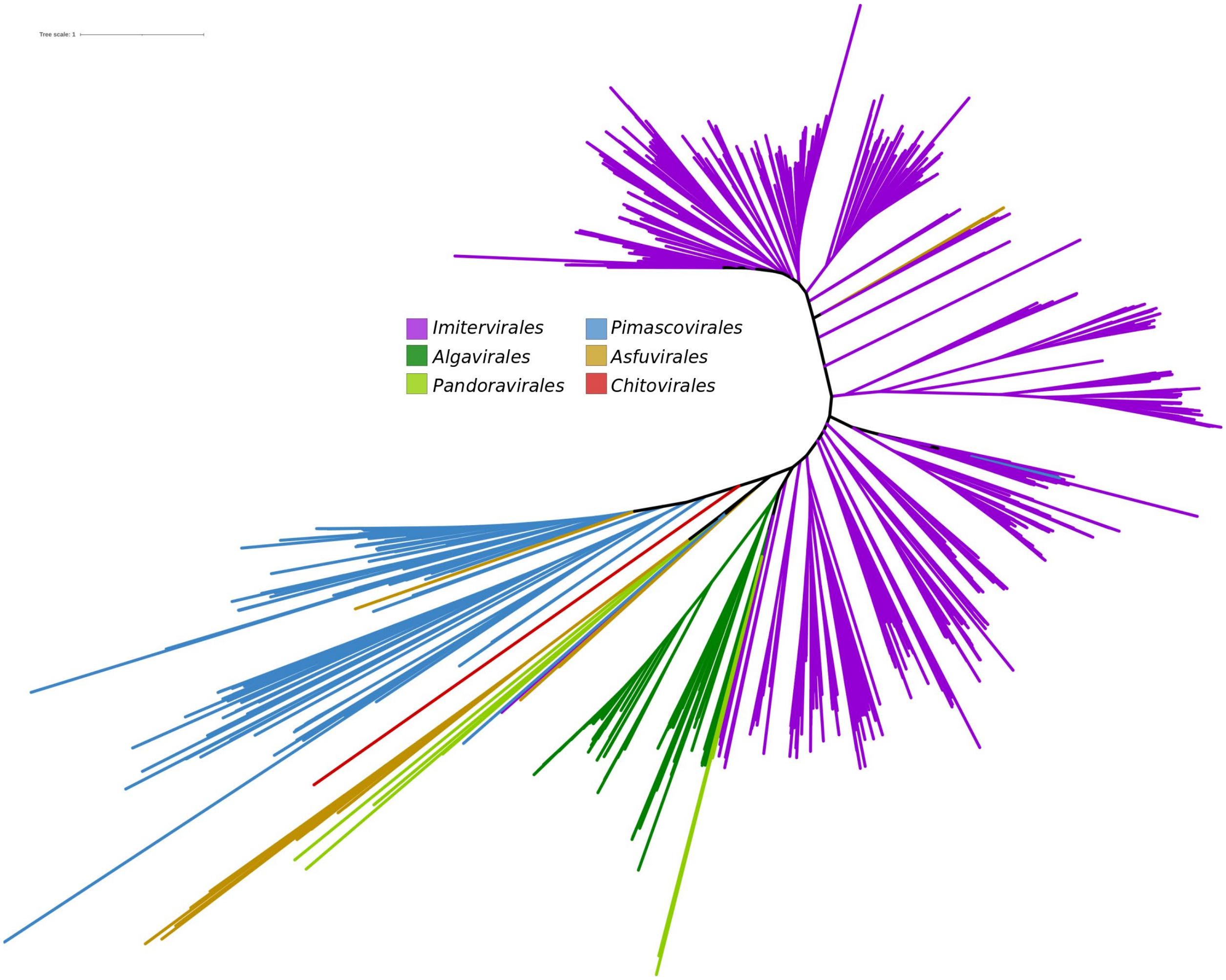

Figure T. Divergent DNA-directed RNA polymerase subunit 5 GVOGm0694 phylogeny.

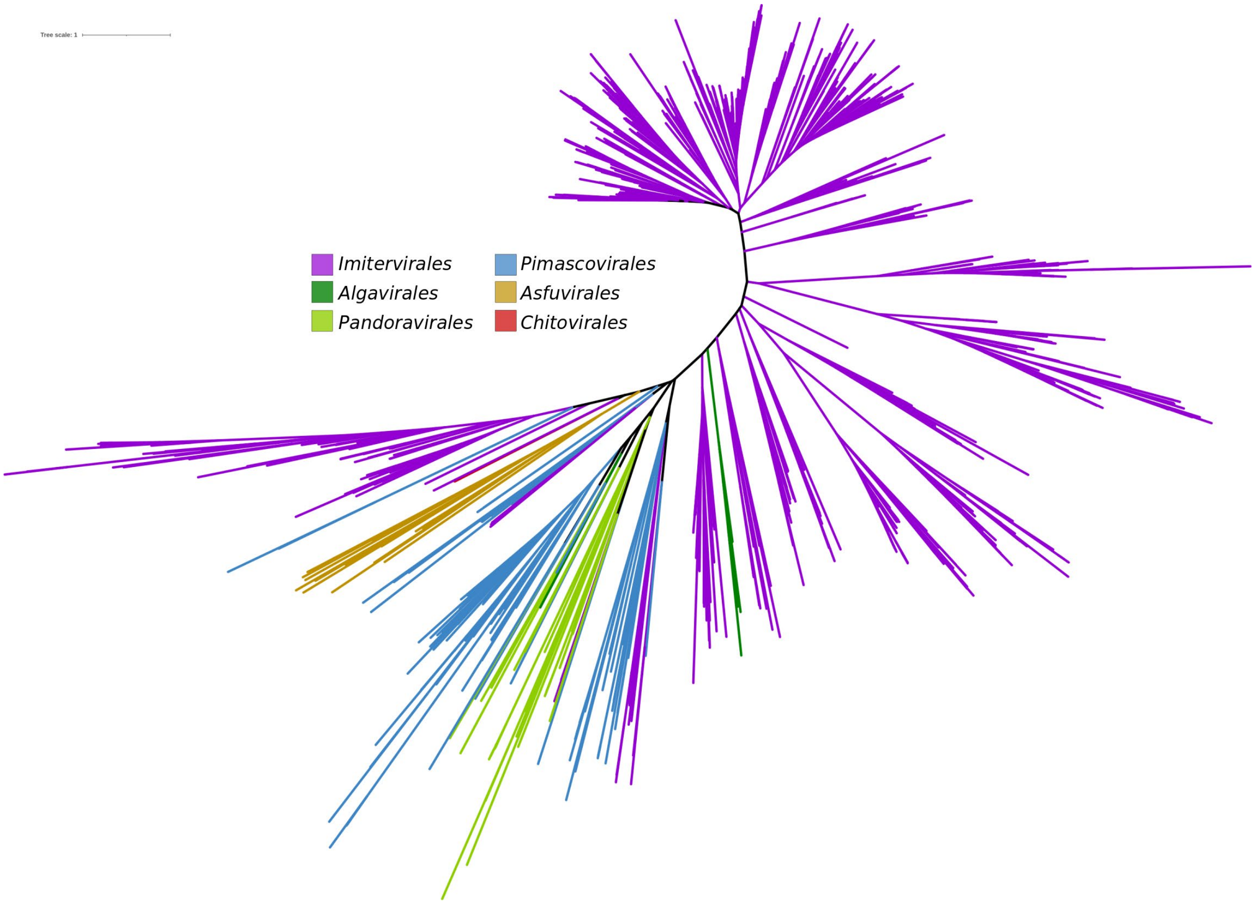

Figure U. Packaging ATPase GVOGm0760 phylogeny.

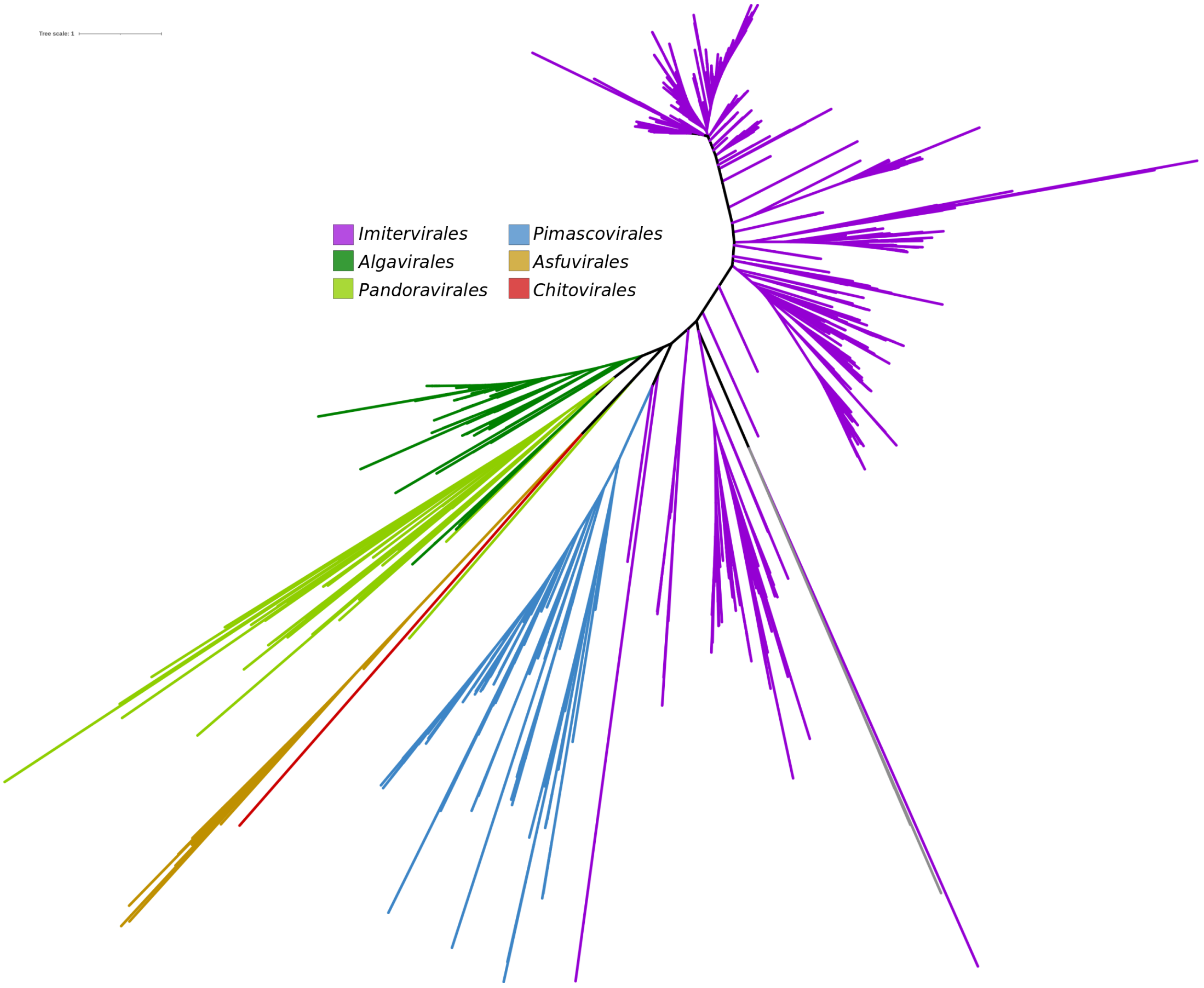

Figure V. Metallopeptidase WLM GVOGm0787 phylogeny.

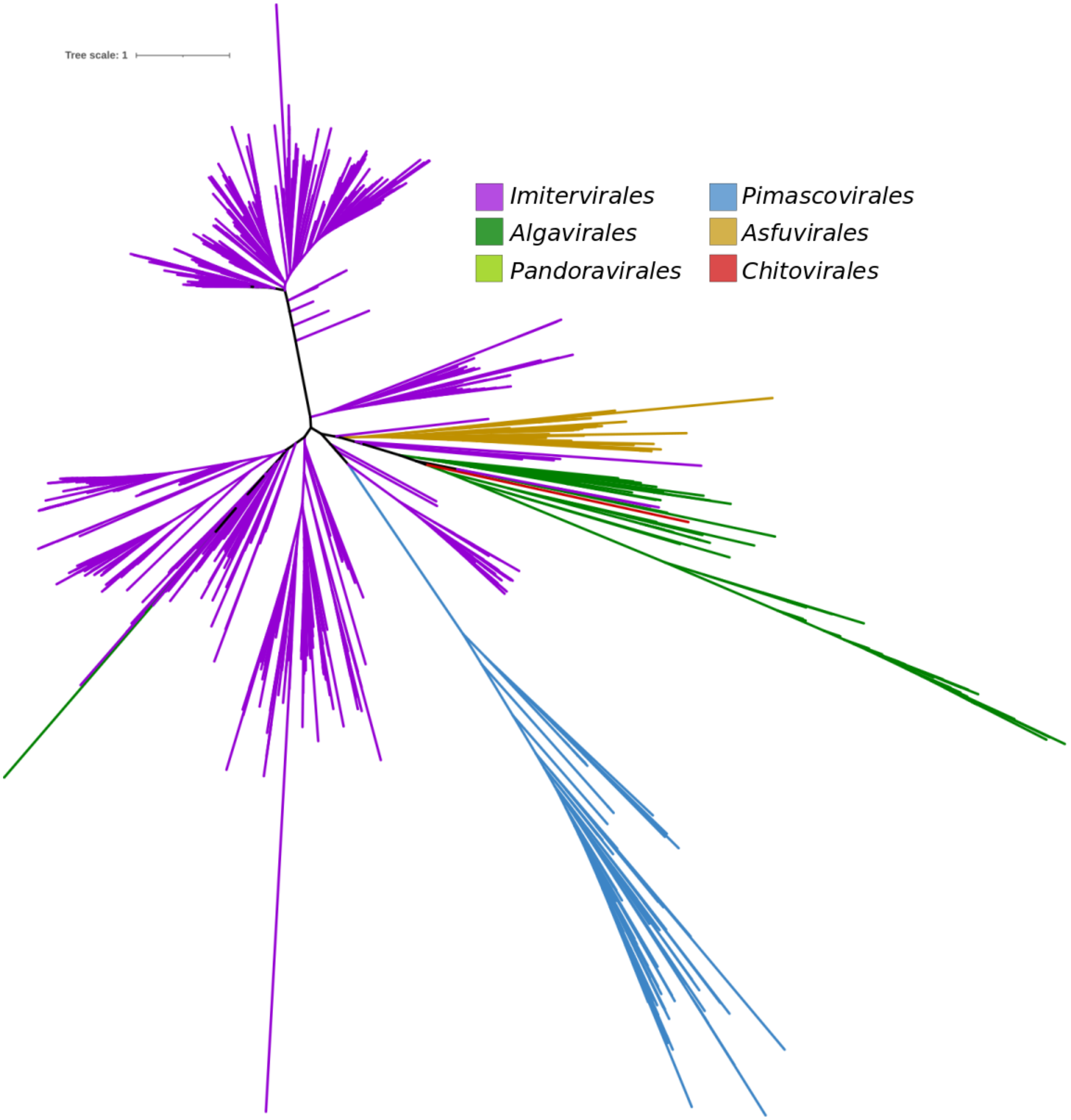

Figure W. Ribonuclease III GVOGm0798 phylogeny.

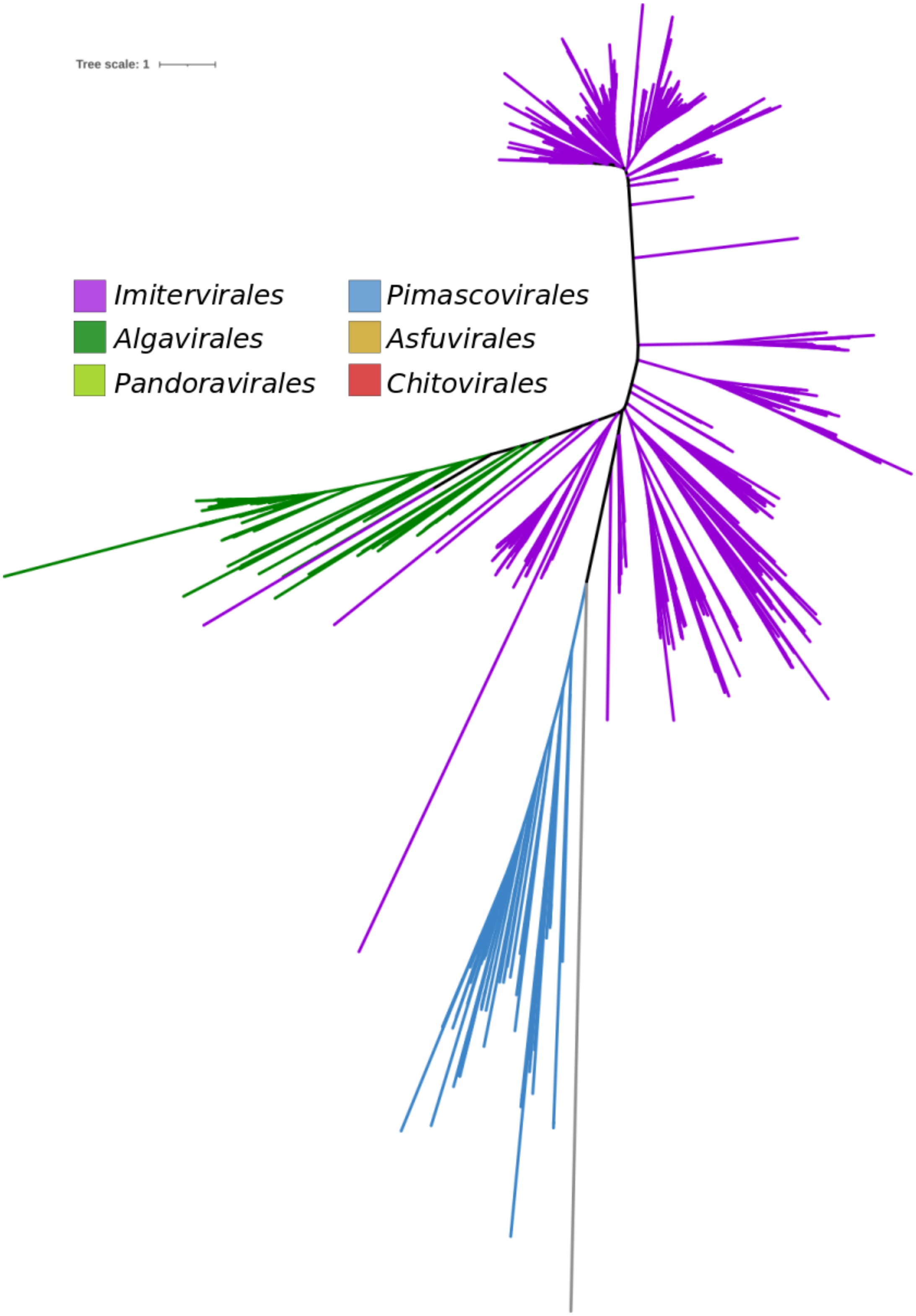

Figure X. Virus Late Transcription Factor 3 VLTF3 GVOGm0890 phylogeny.

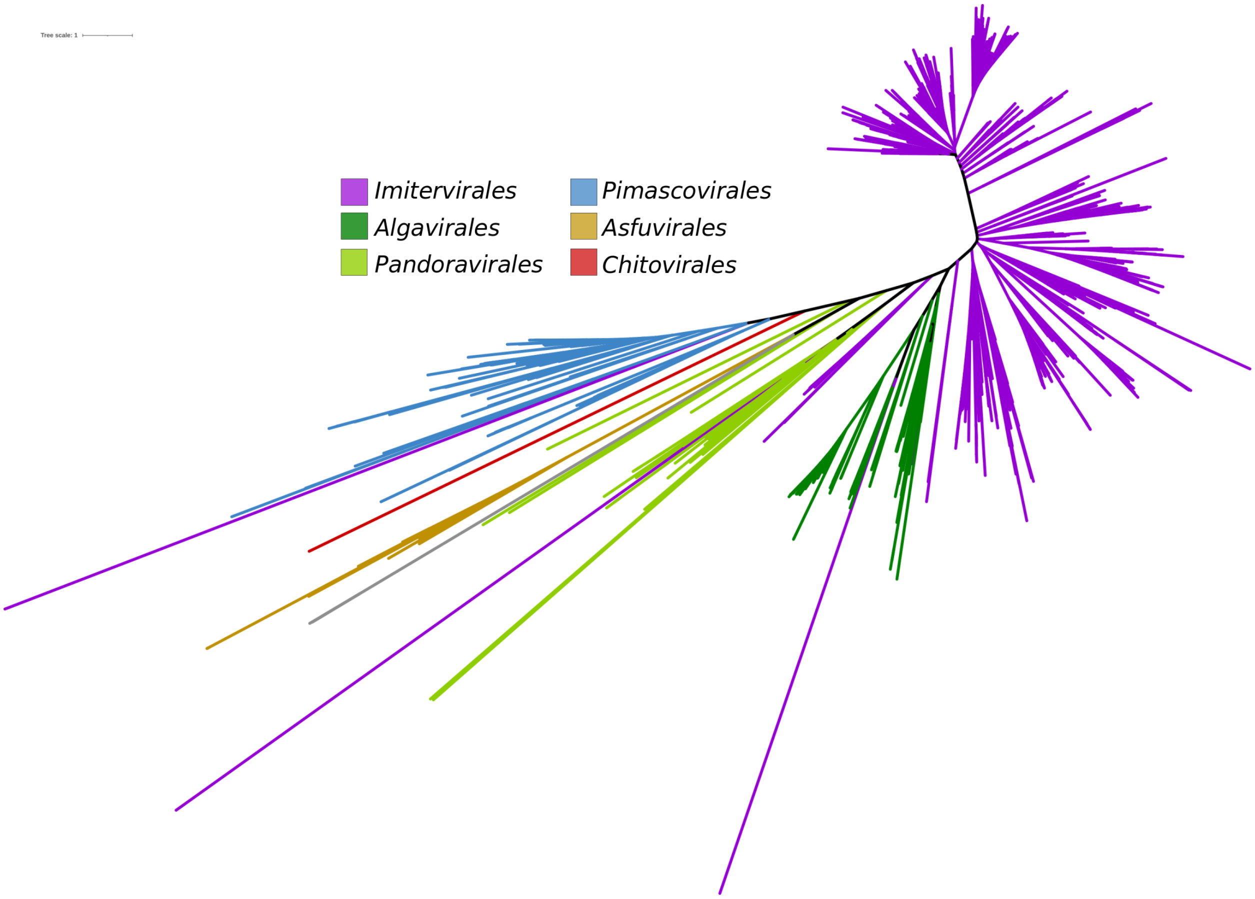

Figure Y. Ribonucleotide reductase small subunit GVOGm1574 phylogeny.

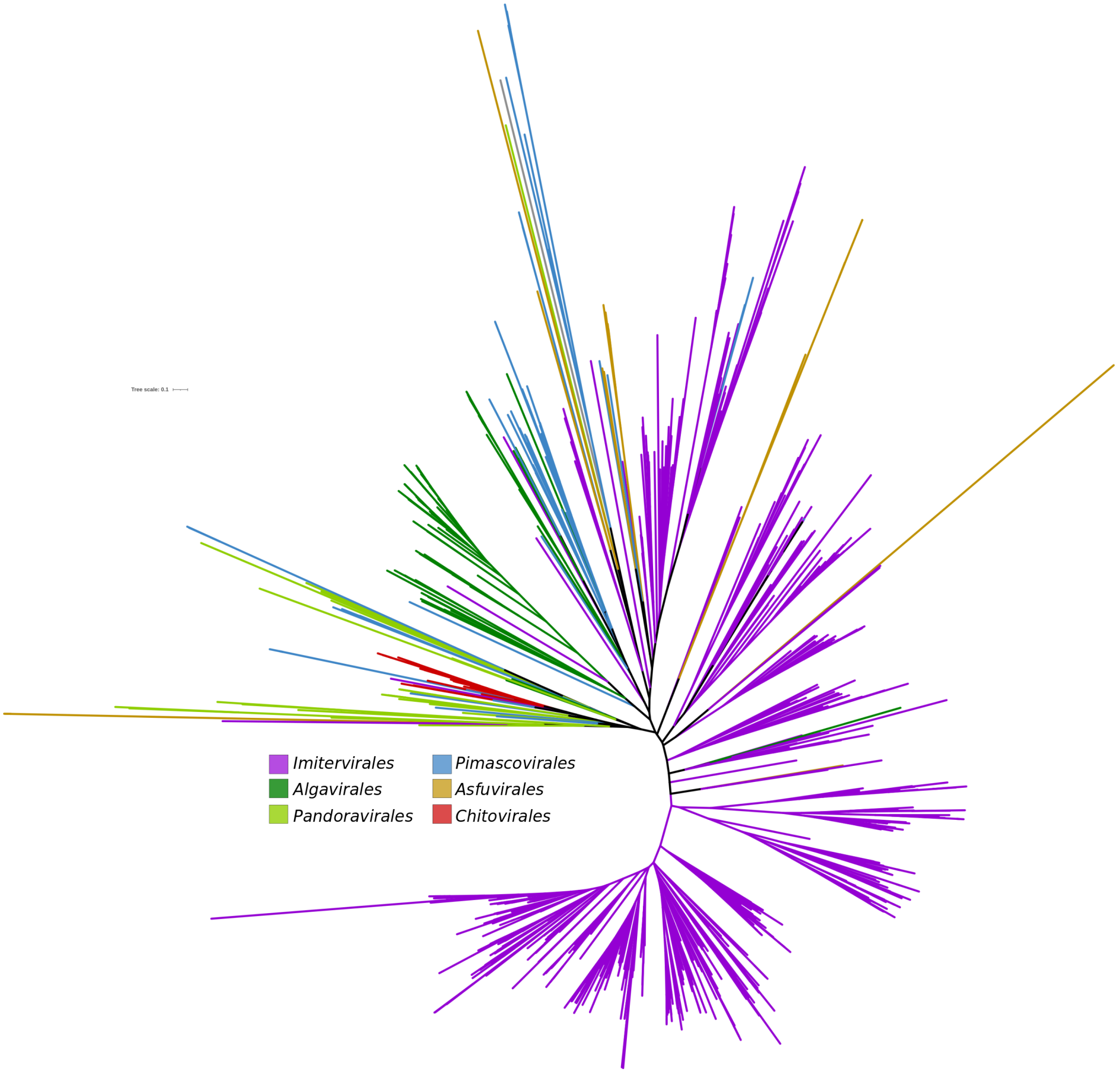

Figure Z. Barchart of source habitats for the *Nucleocytoviricota* families. Full information is provided in S1 Data.

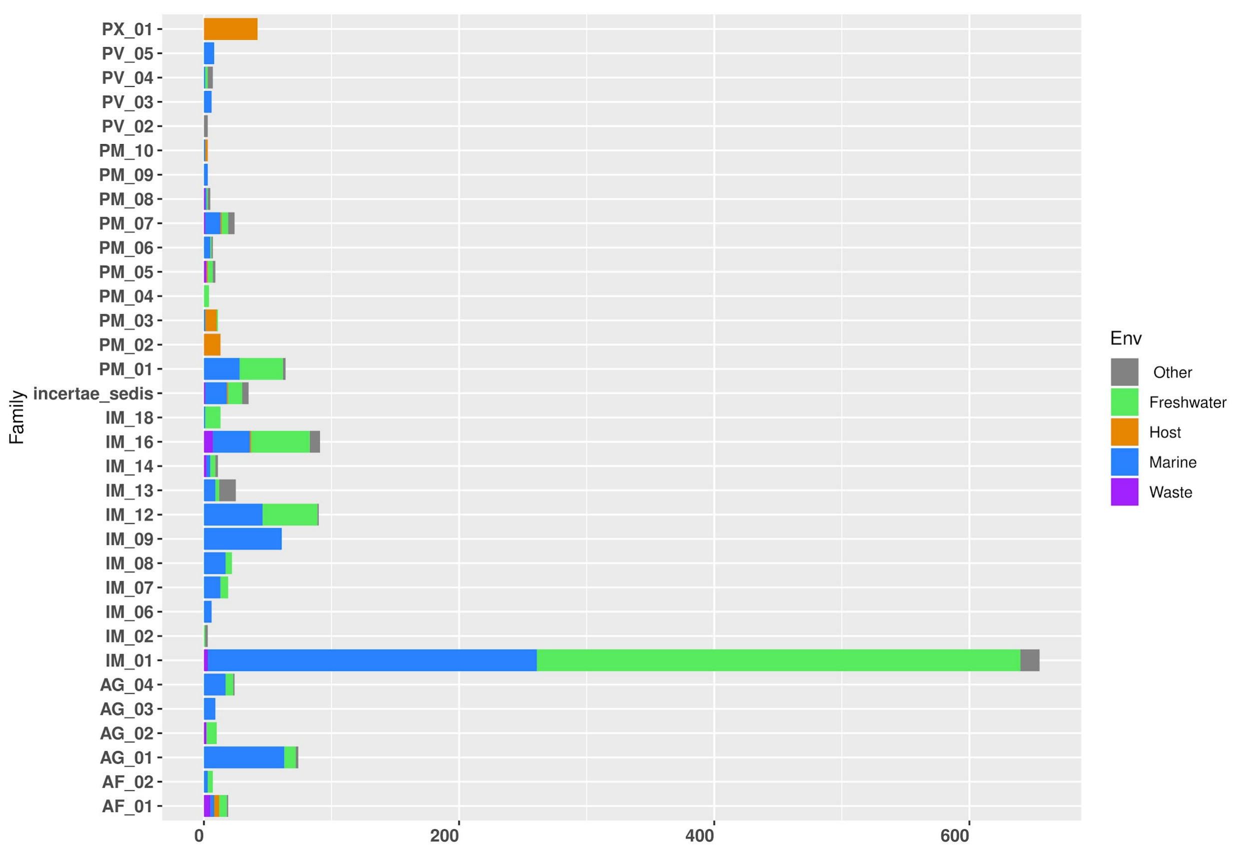

Supplement: S1 Text — Fig A. Major Capsid Protein GVOGm0003 phylogeny. Fig B. Disulfide (thiol) oxidoreductase GVOGm0004 phylogeny. Fig C. Superfamily II helicase GVOGm0013 phylogeny. Fig D. Patatin phospholipase GVOGm0018 phylogeny. Fig E. DEAD/SNF2-like helicase GVOGm0020 phylogeny. Fig F. DNA-directed RNA polymerase subunit beta (RNAPS) GVOGm0022 phylogeny. Fig G. DNA-directed RNA polymerase subunit alpha (RNAPL) GVOGm0023 phylogeny. Fig H. mRNA capping enzyme GVOGm0036 phylogeny. Fig I. DNA polymerase family B GVOGm0054 phylogeny. Fig J. TATA box binding protein (TBP) GVOGm0056 phylogeny. Fig K. Ribonucleoside diphosphate reductase, alpha subunit GVOGm0088 phylogeny. Fig L. D5-like helicase-primase GVOGm0095 phylogeny. Fig M. Uncharacterized, C-terminal domain GVOGm0115 phylogeny. Fig N. Uncharacterized protein GVOGm0152 phylogeny. Fig O. Transcription initiation factor IIB GVOGm0172 phylogeny. Fig P. RuvC, Holliday junction resolvases (HJRs) GVOGm0189 phylogeny. Fig Q. Ubiquitin carboxyl-terminal hydrolase GVOGm0214 phylogeny. Fig R. Proliferating cell nuclear antigen GVOGm0239 phylogeny. Fig S. DNA topoisomerase II GVOGm0461 phylogeny. Fig T. Divergent DNA-directed RNA polymerase subunit 5 GVOGm0694 phylogeny. Fig U. Packaging ATPase GVOGm0760 phylogeny. Fig V. Metallopeptidase WLM GVOGm0787 phylogeny. Fig W. Ribonuclease III GVOGm0798 phylogeny. Fig X. Virus Late Transcription Factor 3 VLTF3 GVOGm0890 phylogeny. Fig Y. Ribonucleotide reductase small subunit GVOGm1574 phylogeny. Fig Z. Barchart of source habitats for the Nucleocytoviricota families. Full information is provided in S1 Data. (PDF) [file pbio.3001430.s001.pdf]
