# Supplementary material for: A phylogenomic framework for charting the diversity and evolution of giant viruses
Source: PLoS Biol. 2021 Oct 27;19(10):e3001430. doi: 10.1371/journal.pbio.3001430 (PMC8575486; doi:10.1371/journal.pbio.3001430)
Supplement: Attachment — Submitted filename: Response_to_Reviewers.docx [file pbio.3001430.s005.docx]

**REVIEWS:**

Reviewer #1: Hiroyuki Ogata. Evolution and ecology of Giant viruses.

Reviewer #2: Virus taxonomy.

Reviewer #3: Giant viruses in soil.

**Reviewer #1:**

Viruses of the phylum Nucleocytoviricota are known for their large genomes, particles, and intriguing biological traits and potential ecological roles. The ICTV taxonomy of this viral group had been incorrect in many aspects for long time until when the recent viral megataxonomy was established in 2020, by a group of leading virologists/taxonomists including one of the authors of this manuscript. However, recent metagenomics has started to produce hundreds to thousands of additional environmental genomes of the Nucleocytoviricota. Consequently, the need for updated phylogenomic studies have re-emerged in order to establish common languages on the taxonomies within the phylum including those uncultivated. As authors correctly pointed out in their manuscript, this updated taxonomic framework is important for ecological and evolutionary analyses of this group of viruses in connection with their relationships with the evolution of cellular organisms. This is very timely and useful work.

The authors compiled 1380 Nucleocytoviricota viral genomes (both isolated and uncultured ones), identified 8863 protein families (named GVOGs), of which they identified 25 GVOGs that are represented widely in the genomes. I have appreciated the next steps that authors took to identify 7 marker genes that are considered as the best choice for phylogenomic analyses, and to objectively delineate clades (class, order, family, and genus level). The choice of marker genes for the phylogenomics is based on the Tree Certainty (TC) scores, while the taxonomic delineation has been performed using a procedure based on the Relative Evolutionary Distance (RAD). To my opinion, this is the best plausible strategy to delineate the taxonomic clades of this viral group. With these methods, the authors partitioned 2 classes, 6 orders, 32 families (this high number is appropriate), and 344 genera within the phylum. The following descriptions of the compositions of each order are easy to read and well-balanced with previous taxonomic proposals. I have nevertheless a few comments that I hope would help to further clarify some points in the current version of the manuscript.

L.33: It would be useful to provide the number of currently recognized viral genera in the phylum Nucleocytoviricota.

**We have modified this sentence to mention the 41 genera within the *Nucleocytoviricota* that are currently recognized by the ICTV (line 35).**

LL.48-54: The citation of the references 9,15,20 appear twice in this part, which makes the logical flow awkward. Probably, the first citation of "9,15,20" would not be needed.

**Thank you for noting this. We have removed the first group of references as recommended.**

L.75: The definition of the word "megataxonomy" may be unclear to some readers (although it is a title of well-cited paper). A few words explaining this jargon would be useful.

**We have clarified this: ‘...the high-level hierarchical taxonomy of all viruses that has recently been adopted (i.e., a “megataxonomy”) ‘ (line 77).**

L.103: "this is likely the case because … multiple copies of MCP, … orthologs from paralogs (Fig. 1a)." To my understanding, for tree reconstructions, the authors chose one sequence for multicopy genes from each genome based on the score against respective HMMs. If it is the case, one MCP at most was chosen from each genome. If this is how data was processed, then, I do not agree to attribute the low TC value for the MCP tree to the existence of paralogs. Because the choice of paralogs or orthologs does not affect the reliability of their tree (if paralogs are mixed, then the tree would not be a species tree but still a gene tree). I guess it is more due to the variable nature of MCPs. Related to this, LL.365-366 for the chose of sequence dataset for individual families is somehow unclear. Does this include only the best hits, or multiple hits if they pass the threshold? A few additional words may help readers to completely understand the procedure.

**We agree this is a complex topic that warrants more discussion.**

**First, it is necessary to clarify that for all phylogenetic analyses, only the best hit for each marker gene was retained. This is the case for phylogenies based on single-gene and multi-gene concatenated alignments.**

**The low TC value for the MCP tree was surprising given that this gene is often used for phylogenetic analysis of *Nucleocytoviricota*. It is true that paralogy itself would not necessarily result in a lower phylogenetic signal, but it is likely that the MCP paralogs experience distinct selective pressure and thus evolve at different rates. If a paralog was included instead of the true ortholog, we would therefore expect that the variable evolutionary rates of the paralogs would decrease the phylogenetic signal. Furthermore, even the evolutionary rate of the true orthologs potentially might be affected by the presence of multiple paralogs. We have added additional text to this section to clarify, and we have also altered our wording to make it clear that this is speculative (lines 98-113).**

**We should also clarify that MCP and RNAPS could still be useful in other contexts, for example, if only complete genomes were included. We included many MAGs in our study in an effort to cover the maximally broad diversity of genomes. Benchmarking was therefore necessary to identify the best set of marker genes to use for this work, but other studies may have reason to use other marker gene sets. For example, one recent study sought to examine the evolutionary relationships of NCLDV compared to eukaryotes, and for this purpose, both RNAP subunits were an appropriate choice (Guglielmini et al., PNAS, 2019).**

**In the Methods, we have added additional text to clarify our approach (lines 381-425). Only the best hit for each marker was retained, and the best hit was required to have a score above a pre-set cutoff. This was done to prevent inclusion of spurious hits into the concatenated phylogenies.**

L.119: The fact that the addition of RNAPS led to the decreased TC may be due to the existence of paralog. In this case, because the goal is to gain the consistency among species trees, the choice of ortholog is needed. RNAPS paralogs was described in<https://journals.asm.org/doi/10.1128/JVI.00230-17>.

**Thank you- we have included this citation in this section.**

L.140: The computation of RED requires the root of the whole tree to my understanding. The authors mentioned that Pox-Asfar group was used to root the tree. However, this would not give the root of the whole phylogenetic tree. How was the root defined? Mid-point? This should be described in the Method section.

**We did not use midpoint rooting. Rather, we rooted the tree between the *Pokkesviricetes* and the *Megaviricetes.* This position defines the root unequivocally and has been adopted in previous studies (Yutin, Wolf, and Koonin, *Virology*, 2014; Koonin and Yutin, *F1000Research*, 2018). Moreover, a recent phylogenomic analysis of the *Varidnaviria* also recapitulated this general topology with other viruses that can serve as an outgroup, albeit with the poxviruses excluded (Figure 4 of Woo et al., *Frontiers in Microbiology*, 2021).**

**Lastly, a detailed phylogenetic analysis of PolB has shown that *Pokkesviricetes* and *Megaviricetes* possess distinct versions of this marker gene (Kazlauskas et al., *Nucleic Acids Research*, 2020). This difference might have arisen due to multiple acquisition events from eukaryotes, or because of orthologous gene displacement in one of these lineages. Regardless, this unique trait distinguishes the two classes and provides further evidence that the root lies between the two. We assessed the placement of the three *Pokkesviricetes incertae sedis* genomes in Figure 2 and confirmed that their PolB copy clusters with the other *Pokkesviricetes*, in support of our placement of these genomes. Phylogenies of individual marker genes also generally show these three genomes clustered with members of the *Pokkesvirictes* (Superfamily II helicase: Figure S3, PolB: Figure S9). We have updated the Methods to include the rationale for root placement (lines 421-425).**

L.154: "including 213 genera with a single representative". Just above this statement, the authors state that clades with two or one members were left as singletons, and for the family count (n=32), the authors excluded the singletons. Consistent counting scheme would be easy to follow.

**In our view, the most novel and impactful finding of our study is the large number of additional families that we delineate within the *Nucleocytoviricota*, and therefore, for this taxonomic rank, we sought to be particularly cautious to avoid over-classification that could occur due to spurious placement of one or a few genomes.**

**Fewer genomes are available for each genus than for each family, and different criteria for delineating this taxonomic level are therefore warranted. Given that only individual genomes are available for many genera, we decided to provide non-redundant names for this taxonomic rank and defer the proposal of new genus names for future studies. Future taxonomic classification will be necessary to clarify the fine-grained nomenclature of *Nucleocytoviricota*.**

L.165: The citation of ref-22 should be ref-47.

**Thank you- this has been changed.**

LL.173-192: The proposed classification scheme does not refer to the Klosneuvirinae, which are also used in several published papers. I suggest the authors to describe their standpoint about this taxonomic group, so that the readers can judge if they wish to continue using this taxonomic group name.

**Thank you for pointing this out. Our study recovered the subfamily *Klosneuvirinae*, consistent with the previous proposal for this subfamily (Schulz et al., Science, 2017). We have added a section describing this, and also discussing the related subfamily *Megamimivirinae* that we also recovered (lines 207-215). The delineation of subfamilies is outside the scope of our taxonomic framework, but we agree it is appropriate to reference some previously-proposed subfamilies.**

L.199-202: Citations of the papers that described the discoveries of individual genes would be needed. For actin, myosin, and kinesin, the citations seem to be<https://doi.org/10.1101/2020.06.16.150565>,<https://doi.org/10.3389/fmicb.2021.683294>, doi: 10.1128/mSystems.00293-21.

**These citations have been added.**

Fig.4: The enrichment of Translation genes in the PM_09 is striking but this is not mentioned in the text. A few words on this may be useful for readers.

**We agree that this is an interesting observation. However, at the moment, this family contains only three genomes, and we are therefore hesitant to make any strong claims regarding the translation-associated genes we found. As more genomes become available, it will be possible to learn more, and we prefer to defer until then for more detailed analysis and discussion.**

L.240-241: In the ref-45, the PolB of medusavirus was proposed to be transferred to eukaryotes, whereas the authors assume a transfer from eukaryotes to medusavirus. These should be distinguished.

**We have revised the wording so that the direction of transfer is left open.**

L.263-272: The authors cite two genomes in the Pimascovirales and three genomes in the Pokkesviricetes. First of all, the branches that correspond to these five genomes should be clearly indicated in the Fig.2. In addition, if the two genomes in the Pimascovirales correspond to the two branches below the Mininucleoviridae, then they look like a family level clade (within the Pimascovirales) instead of an order level clade. If these two genomes form a new order, Mininucleoviridae should be also placed at the order level too. Additional explanation is needed.

**The *Pokkesvirictes insertae sedis* group is marked in Figure 2; we have revised the wording in the text to ensure we refer to this group consistently. We have removed mention of the *Pimascovirales* lineage in this section because we are not proposing a new family for these genomes (lines 288-296). Future phylogenetic work with more genomes will be necessary for further analysis.**

**We have displayed the *Mininucleoviridae* as a polytomy because their placement is highly unstable, likely due to their highly degraded genomes and divergent marker proteins. We retained the family-level designation that has previously been proposed for this group (Sabramanian et al., *mBio*, 2020), but more precise placement within the *Pimascovirales* remains elusive. It cannot be ruled out that future expansion of this group elevates it to the order rank.**

L.333: The author used Prodigal for gene call. How were genes with introns treated? This should be clear as some of the genomes contain many introns.

**When introns are present, Prodigal predicts multiple distinct ORFs, but our custom workflow joins these ORFs together into one protein that is then included in the concatenated alignment. Unfortunately, there is no custom software for gene prediction in NCLDV, and intron-aware programs perform poorly on these viral genomes. We have therefore found that Prodigal is the best available option and that our *post-hoc* procedure for joining proteins adequately deals with them, at least for our purposes here.**

**Reviewer #2:**

The work described in this manuscript to develop a framework for studying the diversity and evolution of large DNA viruses, provides an extremely useful and valuable set of resources (the giant virus orthologous group database along with associated alignments and trees) and data that help to further classify these viruses with members that have either been physically isolated or identified via metagenomic sequencing of environmental samples. As such, it represents an important contribution to the literature. The manuscript will benefit from a careful revision to correct a number of problems, including those detailed below. Major problems include failure to include Table 1, and a misnumbering of tables in the text. In addition, it will be extremely useful to have more of the original data generated in support of this work, available to its readers.

**Thank you for the thorough evaluation of our manuscript. We made revisions in accordance with your comments.**

Lines 54-55: The initial wording of the sentence beginning "The uncertainty of the phylogenetic structure and taxonomy Nucleocytoviricota…" needs rewriting.

**We have changed to “Uncertainty in the phylogenetic relationships within the *Nucleocytoviricota*…”**

Line 83: Table S1 is not a list of genomes. This reference should most likely be to Table S2. Table S2 should provide traditional GenBank accession numbers for all genome sequences when available. When GenBank accessions are not available, the source of the indicated genome sequence (and the provided genome_id) should be identified. I have no idea what some of the provided genome_ids refer to. (I would recommend not concatenating different IDs together.)

**Thank you for pointing this out. Table S1 now contains information on the genomes used, while Table S2 contains information on the GVOGs.**

**We have included a column describing the NCBI accession for the genome, or, if that is not available, the DOI for the publication in which the genomes were presented. When available, we provide NCBI accessions for the assemblies, because that provides a direct link to the genomes themselves, even if multiple contigs are listed. In some cases NCBI assembly IDs were available, in which case we listed nucleotide accessions. In all cases the genomes are publicly available in some form, and also provide all of the genomes on our website (https://faylward.github.io/GVDB/).**

**Nonredundant genome IDs are necessary for several downstream bioinformatic analyses, for which the actual virus taxonomic names are not suitable. The meaning of the names is not particularly important, though for consistency we have tried to retain the same IDs used from the original studies in which these genomes were presented. In some cases we needed to replace special characters such as underscores to retain downstream compatibility with software. We have not concatenated any IDs here, but in some cases NCBI concatenates the assembly and BioProject names together, and in these cases we have retained the original NCBI IDs.**

Line 89: There is no Table 1 provided with the manuscript.

**Apologies- the table was submitted but not combined into the final PDF. We made sure to include it here.**

Lines 311,312: Four sequences are mentioned that are not in an NCBI database. If these sequences are not readily available, they should not be included in this analysis.

**The genomes mentioned here are now all available in NCBI. At the time we conducted this analysis, the genomes had not been incorporated into GenBank.**

Line 90: Table S1 does not provide, as indicated in the text, a "descriptions of all GVOGs".

**We have updated this table to include two tabs; one includes detailed information on the 25 GVOGs assessed for their use as phylogenetic markers, and the second tab includes annotation information for all GVOGs.**

Line 377: There is no Table 1

**Table 1 is now provided (see above).**

Line 387: Are there eight or nine marker gene sets?

**We evaluated 8 different sets of GVOGs.**

Line 401: Now we are at (the final) 7 marker sets. The number of marker gene sets varies according to the analysis providing in figure 3. But in this section of the Methods, it is not clear why these numbers vary from 9 to 8 to 7.

**We evaluated the performance of different combinations of 9 GVOGs. We chose the 9 GVOGs because 1) they are broadly represented across the *Nucleocytoviricota*, and 2) the topology of their individual phylogenetic trees were largely congruent.**

**We tested a total of 8 GVOG combinations (referred to as marker sets), and ultimately we found that a set of 7 GVOGs performed best according to the TC metric. We used a tree derived from this marker set in all subsequent analyses in our study; Figure 2 and 3 are directly derived from this tree.**

**We can understand that the terminology may be somewhat confusing given the different combinations of GVOGs that we tested. We have adjusted the wording of the text throughout the manuscript to clarify exactly what GVOGs were used for the different analyses.**

In the supplementary phylogenetic trees for the set of 25 gene sets, it is very difficult to determine from the provided figures the characterizations of the sets provided in Table S1. For example, for GVOGs 115 and 152, the table indicates that these GVOGS are not represented in viruses belonging to the order Asfuvirales. In the figures for these two GVOG trees, there is also no indication of the presence of poxviruses (order Chitovirales) in the tree. GVOG 152 also appears to not have a representative from the pandoraviruses.

**Thank you for pointing this out- we have revised Table S1 to reflect this. We now have split the existing column of notes into two; one contains all the orders where a GVOG was detected, and the other has general notes on the tree topology for that marker, if applicable.**

In all text and figures, the proposed order pandoravirales should not be capitalized or italicized since it is only proposed and is not an official taxon approved by the ICTV.

**This has been changed.**

Figure 2 and corresponding text: All proposed taxa should, as indicated above, not be capitalized or italicized since they are not official taxa approved by the ICTV.

**This has been changed.**

Figure 2: It would be useful to be able to identify the viruses contributing to each branch of the tree, especially for unlabeled, single genome branches. It is not clear if this information is available or where it might be.

**We now provide a link to an interactive tree on iTOL that users can browse (**[**https://itol.embl.de/shared/faylward**](https://itol.embl.de/shared/faylward)**). Users can view the detailed annotations of our reference tree and also examine the trees produced using different marker gene sets.**

Figure 2: It would be useful to differentiate branches containing viruses derived from culturable isolates vs. viruses assembled from metagenomic data.

**We have updated the figure to include black dots adjacent to families that contain cultured isolates.**

Figure 3a: A supplementary table should be available that provides the complete set of RED values along with, importantly, the identification of the nodes/clades associated with each value.

**All RED values are now available in Table S3. The taxonomic levels for each value are provided, and these names correspond to the ranks outlined in Table S1 and our reference phylogenies on iTOL.**

Along with Table S2, this will provide the data that supports the creation of the proposed new species and families.

**Yes, we agree. The genomes and their placement in our taxonomy are now in Table S1.**

Figure 4. Panels A and B should be labeled on the figure.

**Labels have been added.**

**Reviewer #3:**

Recently several metadata studies (including one by these authors) have identified thousands of new genomes in the Nucleocytoviricota from environmental samples. This current work provides a phylogenetic and more importantly a taxonomic context to interpret and communicate this data. The research uses appropriate state of the art methods. An important aspect is the used of RED values to normalize taxonomic levels to ranges of genetic distances. The manuscript is very well written and will be an important contribution to the field. I only have minor concerns.

**We appreciate your time and the positive feedback.**

In browsing the 25 GVOG trees is the supplemental data S1-25, it was clear that different trees gave very different results. It would be nice to add gene names to the supplemental figures S1-25.

**Thank you for the suggestion- we can see how this would be helpful. We have included the GVOG annotations in the supplemental figure legends.**

It would be good to see the trees associated with the combined marker genes in Fig.1 (maybe I missed these?). How did they vary? This would give a sense of how robust the taxonomy is to any individual gene.

**We have included these benchmarking trees in an iTOL project along with the reference tree (https://itol.embl.de/shared/faylward). All of these trees and their associated alignments are also available on the GVDB along with various other metadata for the genomes used (https://faylward.github.io/GVDB/). The main differences we observed involve placement of the pandoravirales or *Chitovirales*, which is unsurprising given the long branches of these groups. Otherwise the topologies of these trees largely agree.**

Reference in the manuscript the itol tree<https://itol.embl.de/tree/1281731864487941620067021> on the github site.

The authors only suggested names at the phylum, class and order level. All families and genera were given numbers. It would be helpful for communication if names were given at these lower levels too when possible or is the expectation that these will need to be dealt with separately as many genera will need to be renamed as species.

**At this stage we have not proposed any new genus or species names. In the supplemental material, we have propagated all ICTV names when appropriate.**

**Lower-level taxonomic ranks will need to be dealt with separately given the large number of taxonomic names involved.**
